# Sleep Duration and Prostate Cancer Risk in the Southern Community Cohort Study

**DOI:** 10.1002/cam4.71466

**Published:** 2026-01-02

**Authors:** Danica C. Anukam, Roch A. Nianogo, Onyebuchi A. Arah, Paul C. Boutros, Jianyu Rao, Jay H. Fowke, Zuo‐Feng Zhang

**Affiliations:** ^1^ Department of Epidemiology UCLA Fielding School of Public Health Los Angeles California USA; ^2^ Department of Statistics and Data Science UCLA College of Letters and Science Los Angeles California USA; ^3^ Department of Public Health Aarhus University Aarhus Denmark; ^4^ Department of Human Genetics UCLA David Geffen School of Medicine Los Angeles California USA; ^5^ Department of Pathology and Laboratory Medicine UCLA David Geffen School of Medicine Los Angeles California USA; ^6^ Department of Preventive Medicine College of Medicine, University of Tennessee Health Science Center Memphis Tennessee USA; ^7^ Jonsson Comprehensive Cancer Center UCLA Los Angeles California USA; ^8^ Department of Medicine, Center for Human Nutrition UCLA David Geffen School of Medicine Los Angeles California USA

**Keywords:** prostate cancer, racial disparities, SCCS, sleep duration

## Abstract

**Introduction:**

The relationship between insufficient sleep and prostate cancer incidence is unclear. Our goal was to investigate the association of sleep duration, restless sleep, and prostate cancer incidence and aggressiveness, and whether race influences any sleep–prostate cancer association.

**Methods:**

The Southern Community Cohort Study (SCCS) recruited study participants from 12 Southeastern states from 2002 to 2009. The cohort included nearly 35,000 males, predominantly African American (AA, 67%). Sleep exposures were measured via a baseline questionnaire at enrollment, which captured weekday and weekend sleep duration, weighted average sleep duration, and restless sleep. We used Cox proportional hazards models and multinomial logistic regression models to estimate associations between sleep and prostate cancer incidence and aggressiveness.

**Results:**

During follow‐up (median 10.9 years), 1345 men developed prostate cancer. Shorter sleep duration (< 6 h), in comparison to optimal duration (7–8 h), was suggestively associated with a decreased risk of prostate cancer in the overall cohort (adjusted hazard ratio (HR) = 0.83, (95% confidence interval (CI): 0.68–1.01) for sleep average; HR = 0.79, 95% CI: 0.65–0.95 for sleep weekdays; and HR = 0.81, 95% CI: 0.66–0.99 for sleep weekends), and among Black men (HR = 0.77, 95% CI: 0.62–0.97 for sleep average; HR = 0.76, 95% CI: 0.61–0.94 for sleep weekdays; and HR = 0.74, 95% CI: 0.59–0.94 for sleep weekends) when stratified by racial groups. Sleep duration and restless sleep were not associated with prostate cancer aggressiveness overall.

**Conclusion:**

Shorter sleep duration was suggestively associated with a decreased risk of prostate cancer, but sleep duration was not associated with aggressive prostate cancer. Stratified analysis suggests a reduction of prostate cancer risk among Non‐Hispanic Black men with < 6 h of sleep, compared to those sleeping 7–8 h. This is an intriguing finding, and further research is needed to assess the impact of confounding factors on racial differences in prostate cancer development.

## 1 Introduction

Prostate cancer is the most common cancer among men in the United States (US) [1]. The projected new cases of prostate cancer in the US in 2025 are 313,780 [1]. Human sleep can be defined as an individual's behavior and physiological changes in the brain as it shifts between sleep and wakefulness [2]. Sleep plays pivotal roles in several physiologic functions including cognition, development, performance, psychological conditions, and disease [3]. Sleep health promotes overall well‐being and is multifaceted, and while getting the right amount of sleep is critical, other dimensions of sleep are as important including sleep efficiency or continuity, timing, alertness, and quality/satisfaction [4]. Insufficient sleep and poor sleep quality have emerged as public health concerns. In a joint task force statement by the Sleep Research Society (SRS) and American Academy of Sleep Medicine (AASM), their first goal was to address the impact and health of sleep deficiency and circadian dysfunction [5].

Current literature on sleep duration and prostate cancer has been mixed, and previous studies have been inconsistent with the associations between sleep and prostate cancer risk. A few studies found an increased prostate cancer risk with shorter sleep duration or sleep disturbances [6, 7, 8, 9]. Most of the studies on sleep duration and prostate cancer risk found no consistent association [10, 11, 12, 13, 14, 15].

Epidemiologic studies have investigated other aspects of sleep, including restless sleep, sleep disorders, sleep medication, sleep quality, and chronotype. When it comes to associations between sleep disorders, sleep medication, sleep quality, and prostate cancer incidence, results have been unclear. Some studies reported poor sleep quality having an association with an increased prostate cancer risk [16]. In one particular study, Freeman et al. reported that higher wakefulness after sleep onset was related to increased risk of prostate cancer [17]. In another study, Cordina‐Duverger et al. reported that a higher duration of sleep medication was associated with an increased risk of prostate cancer [18]. Other studies have reported that men who suffer from sleep disorders had a higher risk of prostate cancer [9, 19]. However, some studies showed no association between sleep quality and prostate cancer risk [10, 11, 15, 20]. A few studies analyzed the association between night shift work and prostate cancer risk and determined that there was an association [14, 21, 22]. The sleep exposure assessments for the prior studies varied and thus may partly explain observed associations across studies for sleep and prostate cancer incidence.

In addition to the inconsistent findings, there is limited literature on sleep and prostate cancer among black men. African American/black men suffer disproportionately from prostate cancer. In fact, African American/black men have a 68% higher incidence rate of prostate cancer and a more aggressive type than non‐Hispanic white men [1]. While there have been some prospective epidemiological studies examining sleep duration and restless sleep and prostate cancer incidence, none of these prior cohort studies investigated the association in a predominantly non‐Hispanic black cohort. Thus, based on published medical knowledge, this will be one of the early studies that examines the relationship between sleep duration and restless sleep and prostate cancer incidence in a predominantly non‐Hispanic black male cohort. This study aimed to estimate the magnitude of the association between sleep duration and restless sleep and overall prostate cancer incidence. In addition, we seek to determine if sleep predicts a certain diagnosis of prostate cancer (aggressiveness and non‐aggressiveness). This study also aimed to evaluate the racial disparities of the association between sleep characteristics and prostate cancer, stratified by non‐Hispanic black and white men.

## 2 Materials and Methods

### 2.1 Study Design and Study Population

SCCS is a population‐based prospective cohort study and has been discussed elsewhere [23, 24]. In brief, study participants were recruited from 12 Southeastern states (Alabama, Arkansas, Florida, Georgia, Kentucky, Louisiana, Mississippi, North Carolina, South Carolina, Tennessee, Virginia, and West Virginia), and recruitment began in March 2002 through September 2009. The study participants were predominantly African American (67%) and nearly 35,000 male participants were enrolled. Most participants were recruited from community health centers, and they were interviewed at baseline using a computer‐assisted interview by a trained interviewer. The remaining participants were recruited by mail from the general population. At baseline, questions including demographic characteristics, sleep, diet, family history of cancer, medical history information such as prostate cancer screening via prostate‐specific antigen (PSA) test, and medication use, employment status, home environment, and lifestyle factors including physical activity, smoking status, and alcohol consumption were asked to eligible participants.

For the current study, SCCS state cancer registries reporting was completed through 12/31/2016. We examined the SCCS dataset for all males at baseline with the following exclusion criteria: men reporting having prostate cancer prior to baseline (*n* = 598). After exclusion, our analytic sample was 33,568 men, with 22,437 non‐Hispanic Black men, 9609 non‐Hispanic White men, and 1215 men of other racial groups.

### 2.2 Exposure: Sleep Assessment

Sleep duration is the first exposure variable. Sleep duration was asked in a questionnaire at baseline and at follow‐up 1 (2008–2013), but in our study, we focused on baseline only. At baseline, SCCS evaluated sleep duration by asking participants: “How many hours do you typically sleep in a 24‐hour period?” This question was asked for weekdays and for weekends separately, using full‐hour increments. The weighted average sleep duration of weekdays and weekends per 24 h was then calculated as follows: [(weekday sleep duration × 5) + (weekend sleep duration × 2)/7], in which sleep duration was indexed to the closest hour and rounded to a decimal place. Sleep duration (weekday, weekend, and weighted average) was classified into five categories: < 6 h/day (short sleep), 6 h/day, 7–8 h/day, and ≥ 9 h (long sleep), and 7–8 h/day was used as the reference group. Participant responses who did not know or refused to answer the question were recoded as missing.

Restless sleep is the other exposure variable. Participants were asked at baseline how they were feeling during the past week: “my sleep was restless” with the following options: “rarely or none of the time,” “some of the time,” “much of the time,” and “most or all of the time.” “Rarely or none of the time” was the reference group. Participants with “Refuse” or “Don't Know” responses were recoded as missing.

### 2.3 Outcome: Prostate Cancer Assessment

Study outcomes included overall prostate cancer incidence, prostate cancer aggressiveness, and prostate cancer non‐aggressiveness. To acquire information on incident prostate cancer, linkages were mainly from the 12 state cancer registries, and a small number of cases were determined through the National Death Index. For follow‐up data on cancer incidence, data linkage and processing were utilized with the 12 state cancer registries.

Information on prostate cancer aggressiveness and non‐aggressiveness was available for a subset of men who were diagnosed with prostate cancer. The aggressiveness of prostate cancer was based on the Gleason score at diagnosis. We defined aggressive prostate cancer as Gleason score ≥ 8 and non‐aggressive prostate cancer as Gleason score < 8.

### 2.4 Covariates: Confounders and Effect‐Measure‐Modifiers

Based on previous literature and a priori knowledge, we considered a variety of covariates that may influence the relationship between sleep characteristics and prostate cancer. These potential confounders include the following: age at enrollment (40–49, 50–59, 60–69, 70–79 years), enrollment source (community health center (CHC), general population), race/ethnicity (non‐Hispanic white, non‐Hispanic black, other), educational attainment (< 12 years, graduated high school/training/GED, some college or junior college, graduated college or beyond), annual household income (< $15,000, $15,000–$24,999, $25,000–$49,999, ≥ $50,000), BMI (< 18.5, 18.5–< 25.0, 25.0–< 30.0, ≥ 30.0 kg/m^2^), currently working (yes/no), smoking status (never, former, current), alcohol drinking status (non‐drinkers, former drinkers, current drinkers), total physical activity (MET‐hours/day‐continuous), history of diabetes, depression, hypertension, COPD, asthma, stroke (yes/no for each), and benign prostate hyperplasia (BPH) (yes/no). Race/ethnicity was used to assess effect‐measure modification.

### 2.5 Statistical Analysis

To characterize the population, lifestyle, demographic, and socioeconomic variables were included. Means and standard deviations were presented for continuous variables, and frequencies and percentages were presented for categorical variables. To evaluate the association between sleep duration and restless sleep and overall prostate cancer in the analytic sample, Cox proportional hazards regression models were utilized to calculate hazard ratios and 95% confidence intervals to estimate incidence. If cases were identified through the National Death Index, the date of death was utilized as a surrogate for the diagnosis date. The proportional hazards assumption was evaluated by Schoenfeld residuals and was considered met. In the Cox models, follow‐up time was the time scale. The participants were followed from the date of enrollment until the date of prostate cancer diagnosis, death, or December 31, 2016 (the date through which all cancer registries reported having complete data), whichever came first.

In our overall cohort analyses, we first fitted a minimally adjusted model that included age at enrollment and race/ethnicity, and then a fully adjusted model with additional covariates. For analyses stratified by race/ethnicity, we first fitted a minimally adjusted model of enrollment age and a fully adjusted model with covariates.

To evaluate whether sleep predicts the initial diagnosis of prostate cancer (no prostate cancer, aggressive prostate cancer, and non‐aggressive prostate cancer), odds ratios (ORs) and 95% confidence intervals (95% CIs) were calculated using multinomial logistic regression models. These models were used to test whether sleep predicts a certain diagnosis of prostate cancer (aggressive type and non‐aggressive type) relative to no prostate cancer.

To assess effect modification by race/ethnicity, subgroup analyses were conducted such that we stratified the cohort into two groups: non‐Hispanic black and non‐Hispanic white men and evaluated the associations of sleep with prostate cancer risk and aggressiveness in each subgroup. A Wald test was performed to assess the significance of the sleep–race/ethnicity interaction term. All tests and models were conducted on non‐missing data, and missing values of exposure variables and covariates (ranging from 0.9% to 3.6%) were handled via complete‐case analyses.

Sensitivity analyses were performed to compare data when missing data is dealt with using multiple imputation for overall prostate cancer incidence. We ran multiple imputations under the assumption that data were missing at random. For the imputation step, 30 complete datasets were generated. To obtain a set of parameters, each dataset was analyzed individually and combined into overall estimates using Rubin's rule. For the imputation step, 17 covariates were included. Another sensitivity analysis we conducted was to eliminate men diagnosed with prostate cancer within 2 years of follow‐up for overall prostate cancer incidence to account for potential reverse causality. Finally, another sensitivity analysis we conducted was to adjust four additional covariates in our fully adjusted models (Healthy Eating Index (HEI)‐2010 (continuous), family history of prostate cancer including father and brother (yes/no), prostate screening by PSA test (yes/no), and digital rectal exam (DRE) (yes/no)).

Tests for trend for sleep duration were executed, and continuous *p* for trend was calculated by treating categorical sleep duration in the model as a continuous variable. All statistical significance tests were two‐sided. All statistical analyses were performed using SAS statistical software (version 9.4; SAS Institute Inc., Cary, NC).

## 3 Results

### 3.1 Baseline Characteristics of the Study Population

Selected baseline characteristics of the 33,568 participants for the overall study population, including 67.5% Non‐Hispanic black men (*N* = 22,437) and 28.9% Non‐Hispanic white men (*N* = 9609) with a mean age of enrollment being 51.8 years, are presented in Table 1 and stratified by race/ethnicity subgroups. In the analytic sample of the 33,568 participants, most participants were between the ages of 40 and 59, had a low annual household income (< $25,000), and were current drinkers. When stratified by black and white men, over half of black men reported currently smoking, and 60% reported having a household income of < $15,000. Among white men, over half reported not currently working, and about 25% reported having a history of depression. At baseline, 14.3% of the men reported sleeping < 6 h (shorter sleep duration), and 18.0% reported sleeping ≥ 9 h (long sleep duration) for sleep average with somewhat similar proportions for sleep weekday and weekend. Furthermore, 12.9% of men reported that their restless sleep was most or all of the time (Table 1). A more detailed Table 1 with all covariates can be found in Table S1a. The missing data for each sleep trait of the overall study population was < 3.6%, and the frequency and percentage of missing values in the exposure variables and covariates can be found in Table S1b.

**TABLE 1:** Selected baseline characteristics of men, overall and by race in the SCCS study, 2002–2009.

Baseline characteristic	Full cohort (*N* = 33,568)	Non‐Hispanic Black men (*N* = 22,437)	Non‐Hispanic White men (*N* = 9609)
	*N* (%)	*N* (%)	*N* (%)
Enrollment age (years)			
40–49	15,478 (46.1)	11,304 (50.4)	3545 (36.9)
50–59	11,837 (35.3)	7092 (35.2)	3385 (35.2)
60–69	5002 (14.9)	2611 (11.6)	2114 (22.0)
70–79	1251 (3.7)	620 (2.8)	565 (5.9)
Annual household income			
< $15,000	17,714 (54.1)	13,151 (60.0)	3957 (42.0)
$15,000–$24,999	6565 (20.1)	4685 (21.4)	1603 (17.0)
$25,000–$49,999	4627 (14.1)	2761 (12.6)	1622 (17.2)
≥ $50,000	3826 (11.7)	1331 (6.1)	2239 (23.8)
BMI (kg/m^2^)			
< 18.5	386 (1.2)	284 (1.3)	85 (0.9)
18.5–< 25.0	10,698 (32.5)	7698 (35.0)	2635 (27.4)
25.0–< 30.0	11,762 (35.8)	7699 (35.0)	3523 (37.1)
≥ 30.0	10,053 (30.6)	6313 (28.7)	3255 (34.3)
Currently working			
No	19,811 (60.3)	13,504 (61.5)	5537 (58.4)
Yes	13,057 (39.7)	8464 (38.5)	3951 (41.6)
Smoking status			
Never	7658 (23.3)	4893 (22.2)	2406 (25.5)
Former	8097 (24.7)	4543 (20.6)	3141 (33.3)
Current	17,060 (52.0)	12,577 (57.1)	3882 (41.2)
Alcohol drinking status			
Non‐drinkers	2018 (6.2)	1243 (5.7)	671 (7.1)
Former drinkers	8308 (25.4)	5094 (23.3)	2846 (30.2)
Current drinkers	22,343 (68.4)	15,545 (71.0)	5901 (62.7)
History of depression			
No	27,506 (83.1)	19,327 (87.3)	7091 (74.3)
Yes	5604 (16.9)	2804 (12.7)	2456 (25.7)
BPH			
No	30,272 (91.9)	20,864 (94.6)	8157 (86.0)
Yes	2668 (8.1)	1181 (5.4)	1332 (14.0)
Sleep average (h)			
< 6	4661 (14.3)	3168 (14.5)	1254 (13.3)
6	6502 (19.9)	4231 (19.4)	1987 (21.0)
7–8	15,620 (47.9)	9916 (45.5)	5041 (53.4)
≥ 9	5858 (18.0)	4496 (20.6)	1164 (12.3)
Sleep weekday (h)			
< 6	5106 (15.6)	3467 (15.8)	1373 (14.5)
6	7889 (24.1)	5220 (23.8)	2332 (24.6)
7–8	15,394 (46.9)	9895 (45.2)	4852 (51.2)
≥ 9	4411 (13.5)	3335 (15.2)	926 (9.8)
Sleep weekend (h)			
< 6	4644 (14.2)	3218 (14.7)	1182 (12.5)
6	6223 (19.0)	4206 (19.2)	1769 (18.7)
7–8	14,529 (44.4)	9016 (41.2)	4894 (51.7)
≥ 9	7364 (22.5)	5450 (24.9)	1629 (17.2)
Restless sleep			
Rarely or none of the time	11,843 (36.2)	8301 (37.8)	3064 (32.5)
Some of the time	12,960 (39.6)	8929 (40.7)	3555 (37.7)
Much of the time	3726 (11.4)	2156 (9.8)	1369 (14.5)
Most or all of the time	4220 (12.9)	2551 (11.6)	1443 (15.3)

*Note:* Some percentages may not equal 100.0 due to rounding. Missing data not included in percentages.

Abbreviations: BPH, benign prostate hyperplasia; SCCS, Southern Community Cohort Study.

Among 33,568 men, 1345 were diagnosed with prostate cancer over a median follow‐up time of 10.9 (IQR = 4) years, and the mean time between enrollment and prostate cancer diagnosis was 5.9 years. Information on Gleason score was available for 1055 participants (78.4%) diagnosed with prostate cancer. Among the prostate cancer cases with a Gleason score on file, 186 cases were defined as aggressive while 869 cases were defined as non‐aggressive.

### 3.2 Distribution of Baseline Characteristics of the Study Population Stratified by Weighted Sleep Duration

All baseline characteristics by weighted sleep average duration are presented in Table S2a. Compared to men who reported 7–8 h of sleep, a higher percentage of those in the < 6 and ≥ 9 h groups had graduated high school and were obese.

Race‐specific (black and white men) baseline characteristics by weighted sleep duration are presented in Tables S2b and S2c, respectively.

### 3.3 Sleep and Overall Prostate Cancer and Stratified by Race/Ethnicity

The associations of sleep and prostate cancer risk, stratified by race/ethnicity, are summarized in Tables 2 and 3. In the analysis of the entire cohort, we did observe an association between overall prostate cancer risk and < 6 h of sleep for weighted, weekday, and weekend sleep duration.

**TABLE 2:** Complete analyses: Associations between sleep characteristics at enrollment and overall prostate cancer incidence in the SCCS.

	Cohort *N* = 33,568	Cases *N* = 1345	Age and race/ethnicity adjusted^a^	Fully adjusted^b^
	*N* (%)^c^	*N* (%)^c^	HR (95% CI)	HR (95% CI)
*Sleep duration, h*				
Sleep average				
< 6	4388 (14.4)	122 (10.3)	0.82 (0.69–0.99)	0.83 (0.68–1.01)
6	6094 (20.0)	241 (20.4)	1.02 (0.88–1.18)	1.04 (0.89–1.21)
7–8	14,528 (47.7)	591 (50.0)	1.00 (Ref)	1.00 (Ref)
≥ 9	5480 (18.0)	227 (19.2)	1.06 (0.92–1.23)	1.11 (0.95–1.29)
			*p* trend = 0.04	*p* trend = 0.03
Weekday				
< 6	4785 (15.7)	135 (11.4)	0.81 (0.68–0.97)	0.79 (0.65–0.95)
6	7394 (24.2)	271 (22.9)	0.95 (0.83–1.09)	0.92 (0.80–1.06)
7–8	14,278 (46.7)	612 (51.7)	1.00 (Ref)	1.00 (Ref)
≥ 9	4108 (13.4)	166 (14.0)	0.99 (0.84–1.16)	0.99 (0.83–1.18)
			*p* trend = 0.05	*p* trend = 0.02
Weekend				
< 6	4382 (14.3)	117 (9.9)	0.80 (0.66–0.96)	0.81 (0.66–0.99)
6	5850 (19.1)	229 (19.4)	1.00 (0.86–1.16)	1.04 (0.89–1.21)
7–8	13,482 (44.1)	563 (47.6)	1.00 (Ref)	1.00 (Ref)
≥ 9	6847 (22.4)	274 (23.2)	1.03 (0.90–1.18)	1.05 (0.91–1.21)
			*p* trend = 0.04	*p* trend = 0.07
*Restless sleep*				
Rarely/none of time	11,057 (36.1)	496 (42.1)	1.00 (Ref)	1.00 (Ref)
Some of the time	12,114 (39.6)	452 (38.4)	0.94 (0.83–1.06)	0.92 (0.81–1.05)
Much of the time	3470 (11.3)	107 (9.1)	0.94 (0.77–1.15)	0.93 (0.76–1.16)
Most/all of the time	3955 (12.9)	123 (10.4)	0.90 (0.75–1.10)	0.99 (0.81–1.22)

*Note:* Of the 33,568 participants in the analytic cohort, 2848 were excluded from the fully adjusted models for each exposure due to any missing covariate data. Multiple comparisons were adjusted using the Bonferroni method, and results were not significant.

Abbreviations: 95% CI, 95% confidence interval; HR, hazard ratio.

^a^ HR was adjusted for age at enrollment and race/ethnicity.

^b^ HR was additionally adjusted for enrollment source, education, income, employment status, BMI, depression, alcohol drinking, smoking status, diabetes, hypertension, COPD, asthma, stroke, total physical activity, and BPH.

^c^ The cohort and case numbers reflect the fully adjusted model numbers.

**TABLE 3:** Complete analyses: Associations between sleep characteristics at enrollment and prostate cancer incidence by race in the SCCS (cohort *N* = 32,046).

	Cases (Black) *N* = 984	Cases (White) *N* = 304	Age‐adjusted^a^		Race/ethnicity‐specific (fully adjusted)^b^		
			Non‐Hispanic Black	Non‐Hispanic White	Non‐Hispanic Black	Non‐Hispanic White	*p* interaction
	*N* (%)^c^	*N* (%)^c^	HR (95% CI)	HR (95% CI)	HR (95% CI)	HR (95% CI)	
*Sleep duration, h*							
Sleep average							
< 6	94 (10.6)	24 (9.4)	0.81 (0.66–1.00)	0.86 (0.57–1.32)	0.77 (0.62–0.97)	1.17 (0.74–1.83)	
6	189 (21.2)	45 (17.6)	1.09 (0.92–1.29)	0.83 (0.61–1.14)	1.08 (0.91–1.28)	0.96 (0.69–1.35)	
7–8	419 (47.0)	154 (60.2)	1.00 (Ref)	1.00 (Ref)	1.00 (Ref)	1.00 (Ref)	
≥ 9	189 (21.2)	33 (12.9)	1.07 (0.91–1.27)	1.09 (0.78–1.53)	1.10 (0.93–1.31)	1.13 (0.77–1.66)	0.59
			*p* trend = 0.10	*p* trend = 0.17	*p* trend = 0.03	*p* trend = 0.97	
Weekday							
< 6	107 (12.0)	24 (9.3)	0.82 (0.67–1.00)	0.77 (0.50–1.17)	0.76 (0.61–0.94)	0.99 (0.63–1.55)	
6	209 (23.4)	55 (21.4)	0.98 (0.83–1.15)	0.88 (0.66–1.18)	0.93 (0.78–1.09)	0.97 (0.71–1.33)	
7–8	441 (49.4)	153 (59.5)	1.00 (Ref)	1.00 (Ref)	1.00 (Ref)	1.00 (Ref)	
≥ 9	136 (15.2)	25 (9.7)	0.98 (0.82–1.18)	1.03 (0.72–1.49)	0.97 (0.80–1.18)	0.99 (0.64–1.52)	0.51
			*p* trend = 0.14	*p* trend = 0.16	*p* trend = 0.03	*p* trend = 0.93	
Weekend							
< 6	89 (10.0)	23 (9.0)	0.77 (0.62–0.96)	0.89 (0.58–1.36)	0.74 (0.59–0.94)	1.22 (0.77–1.94)	
6	185 (20.7)	38 (14.8)	1.08 (0.91–1.28)	0.75 (0.53–1.05)	1.09 (0.92–1.30)	0.92 (0.64–1.32)	
7–8	390 (43.7)	156 (60.7)	1.00 (Ref)	1.00 (Ref)	1.00 (Ref)	1.00 (Ref)	
≥ 9	228 (25.6)	40 (15.6)	1.05 (0.90–1.23)	0.96 (0.71–1.31)	1.08 (0.92–1.27)	0.93 (0.65–1.31)	0.27
			*p* trend = 0.06	*p* trend = 0.30	*p* trend = 0.03	*p* trend = 0.51	
*Restless sleep*							
Rarely/none of time	395 (44.2)	85 (33.6)	1.00 (Ref)	1.00 (Ref)	1.00 (Ref)	1.00 (Ref)	
Some of the time	340 (38.1)	103 (40.7)	0.90 (0.78–1.03)	1.11 (0.85–1.44)	0.90 (0.78–1.04)	1.10 (0.82–1.46)	
Much of the time	70 (7.8)	34 (13.4)	0.88 (0.69–1.12)	1.19 (0.82–1.74)	0.86 (0.66–1.11)	1.29 (0.86–1.95)	
Most/all of the time	88 (9.9)	31 (12.3)	0.88 (0.70–1.11)	1.04 (0.70–1.54)	0.94 (0.74–1.20)	1.28 (0.83–1.98)	0.08

*Note:* Among those who are black and white only (*n* = 32,046). Due to small sample size, “Other” race (*n* = 1215) was excluded. Analysis is complete case only (thus observations with missing covariates or exposure of interest were excluded). Of the 22,437 black participants and 9609 white participants in the analytic cohort, 1567 were excluded from the fully adjusted models for each exposure due to any missing covariate data for black men, and 850 were excluded from the fully adjusted models for each exposure due to any missing covariate data for white men. Multiple comparisons were adjusted using the Bonferroni method, and results were not significant.

Abbreviations: 95% CI, 95% confidence interval; HR, hazard ratio.

^a^ HR was adjusted for age at enrollment.

^b^ HR was additionally adjusted for enrollment source, education, income, employment status, BMI, depression, alcohol drinking, smoking status, diabetes, hypertension, COPD, asthma, stroke, total physical activity, and BPH.

^c^ The case numbers reflect the fully adjusted model numbers.

In the analyses stratified by black and white men separately, there was an association between sleep duration and prostate cancer risk among black men. Short sleep duration for weighted sleep average (HR = 0.77, 95% CI: 0.62–0.97), weekdays (HR = 0.76, 95% CI: 0.61–0.94), and weekends (HR = 0.74, 95% CI: 0.59–0.94) was associated with decreased prostate cancer risk among black men. In contrast, for prostate cancer risk among white men, elevated hazard ratios for short sleep duration (< 6 h) versus 7–8 h for weighted average sleep were observed with an HR of 1.17 (95% CI: 0.74–1.83), although the HR was not significant. There was not a clear interaction between sleep duration and restless sleep and race/ethnicity in relation to prostate cancer risk.

### 3.4 Sleep Characteristics and Prostate Cancer Risk, by Prostate Aggressiveness and Non‐Aggressiveness and by Race/Ethnicity

We further examined the relationship between sleep and prostate cancer risk by prostate cancer aggressiveness and non‐aggressiveness via multinomial logistic regression models (Table S3). No sleep characteristics were clearly associated with non‐aggressive prostate cancer risk. However, adjusted ORs were all elevated for weighted sleep duration (< 6 and ≥ 9 h) for aggressive prostate cancer, respectively, although 95% confidence intervals all included null values. Stratified by race/ethnicity, similar patterns were observed for average shorter/longer duration of sleep (< 6 and ≥ 9 h) for aggressive prostate cancer for AA men, but not for White men (Table 4).

**TABLE 4:** Complete analyses: Associations between sleep characteristics at enrollment and prostate cancer incidence, by prostate cancer aggressiveness and race in the SCCS.

Overall PC	Non‐aggressiveness^a^‐Black Men		Aggressiveness^b^‐Black Men		Non‐aggressiveness‐White Men		Aggressiveness‐White Men	
	*n*/*N* *	OR (95% CI)^c^	*n*/*N* *	OR (95% CI)^c^	*n*/*N* *	OR (95% CI)^c^	*n*/*N* *	OR (95% CI)^c^
*Sleep duration, h*								
Sleep average								
< 6	62/2999	0.77 (0.58–1.02)	16/2999	1.01 (0.58–1.77)	16/1187	1.21 (0.69–2.13)	5/1187	1.71 (0.58–5.04)
6	126/4018	1.09 (0.87–1.35)	25/4018	1.06 (0.66–1.70)	29/1853	0.95 (0.62–1.46)	6/1853	1.13 (0.43–2.97)
7–8	268/9434	ref	58/9434	ref	103/4589	ref	15/4589	ref
≥ 9	117/4258	1.07 (0.86–1.34)	30/4258	1.26 (0.80–1.97)	20/1068	1.07 (0.65–1.78)	3/1068	0.85 (0.24–3.01)
		*p* trend = 0.13		*p* trend = 0.54		*p* trend = 0.85		*p* trend = 0.31
Weekday								
< 6	72/3270	0.74 (0.57–0.96)	18/3270	1.00 (0.59–1.71)	16/1295	1.03 (0.59–1.80)	5/1295	1.41 (0.49–4.10)
6	139/4949	0.89 (0.72–1.10)	27/4949	0.90 (0.57–1.43)	36/2180	0.97 (0.66–1.44)	6/2180	0.89 (0.34–2.32)
7–8	288/9385	ref	59/9385	ref	102/4404	ref	16/4404	ref
≥ 9	76/3157	0.83 (0.64–1.08)	25/3157	1.34 (0.83–2.15)	15/838	0.92 (0.52–1.63)	2/838	0.59 (0.13–2.65)
		*p* trend = 0.20		*p* trend = 0.32		*p* trend = 0.86		*p* trend = 0.41
Weekend								
< 6	59/3056	0.75 (0.56–1.01)	16/3056	1.06 (0.60–1.87)	16/1122	1.30 (0.73–2.29)	5/1122	1.94 (0.65–5.81)
6	123/3995	1.11 (0.89–1.39)	26/3995	1.19 (0.74–1.92)	24/1649	0.89 (0.56–1.41)	6/1649	1.29 (0.49–3.45)
7–8	250/8552	ref	51/8552	ref	105/4464	ref	14/4464	ref
≥ 9	142/5155	1.05 (0.85–1.30)	36/5155	1.28 (0.83–1.97)	24/1484	0.85 (0.54–1.34)	4/1484	0.87 (0.28–2.71)
		*p* trend = 0.16		*p* trend = 0.67		*p* trend = 0.37		*p* trend = 0.21
*Restless sleep*								
Rarely/none of time	251/7871	ref	61/7871	ref	58/2816	ref	11/2816	ref
Some of the time	223/8492	0.91 (0.75–1.10)	44/8492	0.78 (0.53–1.16)	70/3258	1.09 (0.76–1.56)	6/3258	0.50 (0.18–1.37)
Much of the time	49/2034	0.89 (0.65–1.22)	10/2034	0.84 (0.43–1.67)	22/1278	1.22 (0.73–2.04)	6/1278	1.43 (0.50–4.08)
Most/all of the time	51/2413	0.82 (0.60–1.12)	14/2413	1.08 (0.59–1.96)	20/1350	1.28 (0.74–2.22)	5/1350	1.18 (0.38–3.64)

*Note:* Analysis is complete case only (thus observations with missing covariates or exposure of interest were excluded). *n* = number of men with event for non‐aggressiveness and aggressiveness columns but *n* = number of men without prostate cancer for “no prostate cancer” reference column; *N* = number of men within each group.

Abbreviations: 95% CI, 95% confidence interval; OR, odds ratio.

* The cohort and case numbers reflect the fully adjusted model numbers.

^a^ Non‐aggressiveness defined as Gleason score < 8.

^b^ Aggressiveness defined as Gleason score ≥ 8.

^c^ OR was adjusted for age at enrollment, enrollment source, education, income, employment status, BMI, depression, alcohol drinking, smoking status, diabetes, hypertension, COPD, asthma, stroke, total physical activity, BPH.

### 3.5 Sensitivity Analyses

Sensitivity analyses were conducted to compare data when missing data was dealt with using multiple imputation for overall prostate cancer (Table S4) and stratified by race/ethnicity (Table S5). Results from multiple imputation yielded similar findings in comparison to complete case analyses. We also undertook sensitivity analyses by excluding prostate cancer cases diagnosed within the first 2 years after sleep assessment, and results remained unchanged for overall prostate cancer (Table S6) and among black and white men when examined separately (Table S7). When we additionally adjusted for PSA screening, DRE screening, HEI‐2010, and family history of prostate cancer in our fully adjusted models, the inferences were consistent for overall prostate cancer incidence and aggressiveness and when stratified by black and white men (Tables S8–S11). We also conducted a sensitivity analysis by additionally adjusting for HEI‐2010 and family history of prostate cancer and not adjusting for PSA screening and DRE screening, and inferences were similar (data not shown).

## 4 Discussion

In this cohort consisting of 33,568 men and 10.9 (median) years of follow‐up, we observed associations between shorter sleep duration (< 6 h) and prostate cancer in the whole cohort. While we hypothesized that there would be a positive association between sleep duration and prostate cancer risk, in our study, however, shorter sleep duration was inversely associated with the risk of overall prostate cancer when all men were combined and among AA men. Interestingly, restless sleep “most or all the time” was associated with an increased risk of prostate cancer among White men but not Black men, although the confidence intervals included the null. Further analyses stratified by aggressiveness of prostate cancer showed that adjusted ORs were elevated for sleep duration (< 6 and ≥ 9 h) for aggressive prostate cancer respectively, but not for non‐aggressive prostate cancer, although 95% confidence intervals included null values. Similar patterns were observed among AA men for aggressive prostate cancer.

There are several dimensions to sleep measurements, including sleep duration, efficiency, satisfaction/quality, alertness, and timing [4]. For example, sleep duration consists of the total amount of sleep in 24 h; sleep efficiency is the concept of easily falling asleep and returning to sleep, such as sleep latency; and satisfaction/quality entails the assessment of “poor” or “good” sleep [4]. Current literature on one aspect of the sleep dimension, sleep duration, and prostate cancer incidence has been mixed. One study found an association between short sleep duration and increased prostate cancer risk [6]. However, several studies found no significant association [8, 10, 11, 14, 15, 17, 18, 25]. Liu et al. conducted a meta‐analysis of six prospective cohort studies and found that sleep duration was not associated with the risk of prostate cancer [13].

We found that short sleep duration was suggestively associated with a decreased risk of overall prostate cancer in the whole cohort. This finding was surprising as it did not support our hypothesis. There has been one prostate cancer study with a similar finding. In a large prospective cohort of US health professionals, Markt et al. found that men who slept ≤ 6 h compared to 7–8 h had a reduced risk of overall prostate cancer (RR = 0.90, 95% CI = 0.83–0.97) [12]. There have been a few epidemiological studies with similar findings but that were not significant. In another prospective study from Sweden, Markt et al. found that sleeping ≤ 5 h in comparison to 8 h was associated with decreased prostate cancer risk (HR = 0.92, 95% CI = 0.66–1.28) [10].

In this study, there was no clear evidence of interactions of sleep characteristics with race/ethnicity. However, shorter sleep duration for weighted weekdays and weekends was associated with decreased prostate cancer risk among AA men only, which was contrary to our hypothesis. Our study population consisted of predominantly AA men, while other studies were predominantly European and Asian populations. Compared to white adults, AA adults are nearly twice as likely to report short sleep duration [26]. In addition, AAs are more likely to report being long sleepers and tend to take longer to fall asleep [26]. In the Multi‐Ethnic Study of Atherosclerosis (MESA), Chen et al. found that blacks were 4.95 times more likely than non‐Hispanic white adults to have a sleep duration of < 6 h and 1.57 times more likely to have poor sleep quality [27]. Our observation of the association between short sleep duration (< 6 h) and decreased prostate cancer risk among AA men is intriguing because it contrasts with prior knowledge that sleep deprivation may potentially increase cancer risk.

Sleep is very critical for maintaining testosterone levels, which is indicated in prostate cancer growth. Short sleep duration has been associated with reduced testosterone levels [28]. Su et al. conducted a meta‐analysis and found that total sleep deprivation reduced male serum testosterone levels [29]. Testosterone is a major androgen in men that is responsible for different physiologic processes such as sleep. Since prostate cancer is androgen‐dependent, lower testosterone levels due to sleep deprivation may reduce cancer risk. Watts et al. analyzed individual men data from 20 prospective studies and found that men with low concentrations of circulating free testosterone may have a reduced risk of prostate cancer [30]. In our study, we found some evidence of a negative association between shorter sleep duration and overall prostate cancer risk for AA men, but a positive association for white men. One potential hypothesis for this finding could be the racial differences of testosterone levels among black and white men. Based on analyses utilizing the National Health and Nutritional Examination Survey (NHANES), Hu et al. concluded that with increasing age, testosterone levels in black men decreased considerably more than in white men [31].

Besides assessing sleep duration, another informative measure of sleep quality is restless sleep. In our study, we did not find consistent associations between restless sleep and prostate cancer. This lack of association is in line with one other study in which Markt et al. found no association between problems falling asleep or staying asleep and prostate cancer risk [12]. However, there have been other previous studies that have found positive associations. Analysis in the UK Biobank found that finding it fairly easy to get up in the morning was positively associated with prostate cancer [19]. Another finding in Wiggins et al. showed that difficulty falling asleep at night was positively associated with high‐grade prostate cancer [20]. Finally, Sigurdardottir et al. found that men with sleep disruption including problems falling asleep and staying asleep had a significantly increased risk of advanced prostate cancer [7]. In our study, we did not find consistent patterns of association between sleep and prostate cancer and prostate cancer aggressiveness. Interestingly, though, when we analyzed the association between sleep duration and prostate cancer aggressiveness, U‐shaped curves were observed such that shorter sleep duration and long duration of sleep were associated with an increased risk of aggressive prostate cancer, indicating that sleep association should consider aggressiveness. Our findings were similar to a case‐control study in France that found a U‐curve shape between sleep duration and risk of aggressive prostate cancer [18]. One possible explanation for the findings of sleep and prostate cancer aggressiveness could stem back to testosterone. In an analysis of a large cohort in Texas, Tu et al. concluded that low total serum testosterone levels were associated with tumor aggressiveness and predicted poor prognosis [32].

This study had several strengths and limitations. There are some strengths for this study. For one, there is a systematic follow‐up to identify incident prostate cancer through mainly linkage to statewide registries as well as information on subtypes of prostate cancer by Gleason score. It is a large prospective study with a large population of black men included in the analysis. Another strength is that we controlled a wide number of potential confounders such as sociodemographic characteristics, physical conditions, and behavioral factors. Other strengths include sleep duration and restless sleep measured before the development of prostate cancer. With strengths, there are limitations. One limitation is that we lacked information that could affect sleep patterns such as sleep medication usage, psychiatric conditions, and sleep disorders. We also focused on a single report of sleep duration and restless sleep, which may not be reflective of a participant's sleep patterns long term. Another limitation is the small sample size when the analyses were stratified by aggressiveness and by race/ethnicity. Thus, associations may have been detected partially due to low case counts. Finally, another limitation is that sleep duration and restless sleep were self‐reported rather than via objective estimations such as actigraphy or polysomnography. For example, in a validation study, Lauderdale and her colleagues found that there was an overestimation of self‐reported sleep duration in comparison with actigraphy‐measured sleep duration, especially among adults with short sleep duration and among African Americans, and a moderate correlation (correlation coefficient 0.45) was found between self‐reported and actigraphy‐measured sleep duration [33].

## 5 Conclusion

In summary, shorter sleep duration was suggestively associated with a decreased risk of prostate cancer. Stratified analysis suggests a reduction of prostate cancer risk among Non‐Hispanic Black men with < 6 h of sleep, compared to those sleeping 7–8 h, which was not observed among White men. Our study is one of the first studies to examine sleep and prostate cancer risk among Black men. The effect of sleep on prostate cancer, especially among Black men, deserves further investigation, and further research is imperative to identify potential mechanisms for these suggestive associations. Future research is needed with comprehensive and clear sleep assessments and objective sleep measurements and other aspects of sleep quality over time.

## Author Contributions

**Danica C. Anukam:** conceptualization (equal), data curation (equal), formal analysis (lead), investigation (equal), methodology (equal), software (lead), writing – original draft (lead), writing – review and editing (equal). **Roch A. Nianogo:** writing – review and editing (equal). **Onyebuchi A. Arah:** methodology (equal), writing – review and editing (equal). **Paul C. Boutros:** writing – review and editing (equal). **Jianyu Rao:** writing – review and editing (equal). **Jay H. Fowke:** writing – review and editing (equal). **Zuo‐Feng Zhang:** conceptualization (equal), data curation (equal), funding acquisition (equal), investigation (equal), methodology (equal), project administration (equal), resources (equal), supervision (lead), writing – review and editing (equal).

## Funding

This work was supported by the National Cancer Institute (Grants CA009142 and U01CA202979) and HEALRISE (HEALth, Racism, Inequities, and Social Epidemiology) Fellowship.

## Ethics Statement

All study participants provided written consent at enrollment, and this study was approved by the Institutional Review Boards of Vanderbilt University and Meharry Medical College.

## Conflicts of Interest

The authors declare no conflicts of interest.

## Supporting information


**Table S1a:** All baseline characteristics of men, overall and by race in the SCCS, 2002–2009.
**Table S1b:** Frequency and percentage of missing values in the variables of interest.
**Table S2a:** All baseline characteristics of men by sleep average in the SCCS, 2002–2009.
**Table S2b:** All baseline characteristics of black men by sleep average in the SCCS, 2002–2009.
**Table S2c:** All baseline characteristics of white men by sleep average in the SCCS, 2002–2009.
**Table S3:** Complete analyses: Associations between sleep characteristics at enrollment and prostate cancer incidence, by prostate cancer aggressiveness in the SCCS.
**Table S4:** Multiple imputation: Associations between sleep characteristics at enrollment and overall prostate cancer incidence in the SCCS.
**Table S5:** Multiple imputation: Associations between sleep characteristics at enrollment and overall prostate cancer incidence by race in the SCCS.
**Table S6:** Associations between sleep characteristics at enrollment and overall prostate cancer incidence in the SCCS.
**Table S7:** Associations between sleep characteristics at enrollment and prostate cancer incidence by race in the SCCS (Cohort *N* = 31,810).
**Table S8:** Complete analyses: Associations between sleep characteristics at enrollment and overall prostate cancer incidence in the SCCS.
**Table S9:** Complete analyses: Associations between sleep characteristics at enrollment and prostate cancer incidence by race in the SCCS (Cohort *N* = 32,046).
**Table S10:** Complete analyses: Associations between sleep characteristics at enrollment and prostate cancer incidence, by prostate cancer aggressiveness in the SCCS.
**Table S11:** Complete analyses: Associations between sleep characteristics at enrollment and prostate cancer incidence, by prostate cancer aggressiveness and race in the SCCS.

## Data Availability

The data underlying this article were obtained through a data access agreement with the Southern Community Cohort Study. The analytic code, code book, and data are available from the corresponding author on reasonable request and in agreement with data access rules set by the Southern Community Cohort Study.
