# Supplementary material for: Sleep Duration and Prostate Cancer Risk in the Southern Community Cohort Study
Source: Cancer Med. 2026 Jan 2;15(1):e71466. doi: 10.1002/cam4.71466 (PMC12759263; doi:10.1002/cam4.71466)
Supplement: Supplementary file 1 — Table S1a: All baseline characteristics of men, overall and by race in the SCCS, 2002–2009. Table S1b: Frequency and percentage of missing values in the variables of interest. Table S2a: All baseline characteristics of men by sleep average in the SCCS, 2002–2009. Table S2b: All baseline characteristics of black men by sleep average in the SCCS, 2002–2009. Table S2c: All baseline characteristics of white men by sleep average in the SCCS, 2002–2009. Table S3: Complete analyses: Associations between sleep characteristics at enrollment and prostate cancer incidence, by prostate cancer aggressiveness in the SCCS. Table S4: Multiple imputation: Associations between sleep characteristics at enrollment and overall prostate cancer incidence in the SCCS. Table S5: Multiple imputation: Associations between sleep characteristics at enrollment and overall prostate cancer incidence by race in the SCCS. Table S6: Associations between sleep characteristics at enrollment and overall prostate cancer incidence in the SCCS. Table S7: Associations between sleep characteristics at enrollment and prostate cancer incidence by race in the SCCS (Cohort N = 31,810). Table S8: Complete analyses: Associations between sleep characteristics at enrollment and overall prostate cancer incidence in the SCCS. Table S9: Complete analyses: Associations between sleep characteristics at enrollment and prostate cancer incidence by race in the SCCS (Cohort N = 32,046). Table S10: Complete analyses: Associations between sleep characteristics at enrollment and prostate cancer incidence, by prostate cancer aggressiveness in the SCCS. Table S11: Complete analyses: Associations between sleep characteristics at enrollment and prostate cancer incidence, by prostate cancer aggressiveness and race in the SCCS. [file CAM4-15-e71466-s001.docx]

## **Table S1a. All baseline characteristics of men, overall and by race in the SCCS, 2002-2009**

|  | **Full Cohort (n=33,568)** | **Non-Hispanic Black Men (n=22,437)** | **Non-Hispanic White Men (n=9,609)** |
| --- | --- | --- | --- |
| **Baseline Characteristic** | **N (%)** | **N (%)** | **N (%)** |
| Enrollment age (years) |  |  |  |
| 40-49 | 15478 (46.1) | 11304 (50.4) | 3545 (36.9) |
| 50-59 | 11837 (35.3) | 7092 (35.2) | 3385 (35.2) |
| 60-69 | 5002 (14.9) | 2611 (11.6) | 2114 (22.0) |
| 70-79 | 1251 (3.7) | 620 (2.8) | 565 (5.9) |
|  |  |  |  |
| Enrollment source |  |  |  |
| CHC in-person interview | 28071 (83.6) | 20532 (91.5) | 6541 (68.1) |
| General population | 5497 (16.4) | 1905 (8.5) | 3068 (31.9) |
|  |  |  |  |
| Race/Ethnicity |  |  |  |
| Non-Hispanic white | 9609 (28.9) | 0 (0.0) | 9609 (100.0) |
| Non-Hispanic black | 22437 (67.5) | 22437 (100.0) | 0 (0.0) |
| Other | 1215 (3.7) | 0 (0.0) | 0 (0.0) |
|  |  |  |  |
| Educational attainment |  |  |  |
| Less than 12 years | 9845 (29.9) | 7435 (33.6) | 2123 (22.2) |
| Graduated high school, training, or GED | 12800 (38.9) | 9045 (40.8) | 3345 (35.0) |
| Some college or junior college | 5915 (18.0) | 3804 (17.2) | 1836 (19.2) |
| Graduated college or beyond | 4362 (13.3) | 1867 (8.4) | 2247 (23.5) |
|  |  |  |  |
| Annual household income |  |  |  |
| < $15,000 | 17714 (54.1) | 13151 (60.0) | 3957 (42.0) |
| $15,000 – $24,999 | 6565 (20.1) | 4685 (21.4) | 1603 (17.0) |
| $25,000 – $49,999 | 4627 (14.1) | 2761 (12.6) | 1622 (17.2) |
| ≥$50,000 | 3826 (11.7) | 1331 (6.1) | 2239 (23.8) |
|  |  |  |  |
| BMI (kg/m^2^) |  |  |  |
| <18.5 | 386 (1.2) | 284 (1.3) | 85 (0.9) |
| 18.5 - <25.0 | 10698 (32.5) | 7698 (35.0) | 2635 (27.4) |
| 25.0 - <30.0 | 11762 (35.8) | 7699 (35.0) | 3523 (37.1) |
| ≥30.0 | 10053 (30.6) | 6313 (28.7) | 3255 (34.3) |
|  |  |  |  |
| Currently working |  |  |  |
| No | 19811 (60.3) | 13504 (61.5) | 5537 (58.4) |
| Yes | 13057 (39.7) | 8464 (38.5) | 3951 (41.6) |
|  |  |  |  |
| Smoking status |  |  |  |
| Never | 7658 (23.3) | 4893 (22.2) | 2406 (25.5) |
| Former | 8097 (24.7) | 4543 (20.6) | 3141 (33.3) |
| Current | 17060 (52.0) | 12577 (57.1) | 3882 (41.2) |
|  |  |  |  |
| Alcohol drinking status |  |  |  |
| Non-drinkers | 2018 (6.2) | 1243 (5.7) | 671 (7.1) |
| Former drinkers | 8308 (25.4) | 5094 (23.3) | 2846 (30.2) |
| Current drinkers | 22343 (68.4) | 15545 (71.0) | 5901 (62.7) |
|  |  |  |  |
| Total physical activity (MET-hours/day), mean (SD) | 25.4 (22.7) | 26.3 (23.3) | 23.3 (21.0) |
| HEI-2010, mean (SD) | 55.2 (11.5) | 55.0 (11.0) | 55.4 (12.3) |
|  |  |  |  |
| History of diabetes |  |  |  |
| No | 27016 (81.6) | 18108 (81.8) | 7806 (81.8) |
| Yes | 6093 (18.4) | 4024 (18.2) | 1739 (18.2) |
|  |  |  |  |
| History of depression |  |  |  |
| No | 27506 (83.1) | 19327 (87.3) | 7091 (74.3) |
| Yes | 5604 (16.9) | 2804 (12.7) | 2456 (25.7) |
|  |  |  |  |
| History of hypertension |  |  |  |
| No | 16656 (50.3) | 10987 (49.6) | 4932 (51.7) |
| Yes | 16462 (49.7) | 11151 (50.4) | 4616 (48.4) |
|  |  |  |  |
| History of COPD |  |  |  |
| No | 30861 (93.2) | 21039 (95.1) | 8530 (89.4) |
| Yes | 2239 (6.8) | 1090 (4.9) | 1013 (10.6) |
|  |  |  |  |
| History of asthma |  |  |  |
| No | 29567 (89.3) | 19923 (90.0) | 8389 (87.9) |
| Yes | 3546 (10.7) | 2212 (10.0) | 1159 (12.1) |
|  |  |  |  |
| History of stroke |  |  |  |
| No | 31070 (93.9) | 20880 (94.4) | 8870 (93.0) |
| Yes | 2025 (6.1) | 1245 (5.6) | 672 (7.0) |
|  |  |  |  |
| Family history of PC |  |  |  |
| No | 29698 (94.6) | 19926 (95.0) | 8530 (94.0) |
| Yes | 1685 (5.4) | 1045 (5.0) | 548 (6.0) |
|  |  |  |  |
| BPH |  |  |  |
| No | 30272 (91.9) | 20864 (94.6) | 8157 (86.0) |
| Yes | 2668 (8.1) | 1181 (5.4) | 1332 (14.0) |
|  |  |  |  |
| PSA Screening |  |  |  |
| No | 15890 (51.0) | 11260 (53.8) | 3968 (44.5) |
| Yes | 15292 (49.0) | 9664 (46.2) | 4952 (55.5) |
|  |  |  |  |
| DRE screening |  |  |  |
| No | 13346 (40.6) | 9461 (43.0) | 3347 (35.3) |
| Yes | 19562 (59.4) | 12537 (57.0) | 6143 (64.7) |

Note: Some percentages may not equal to 100.0 due to rounding. Missing data not included in percentages

SCCS: Southern Community Cohort Study. CHC: Community Health Center. BMI: body mass index.

SD: standard deviation. MET: metabolic equivalent of task. HEI-2010: Healthy Eating Index. COPD: Chronic obstructive

pulmonary disease. PC: prostate cancer. BPH: benign prostate hyperplasia. PSA: prostate cancer-specific antigen. DRE:

digital rectal examination.

| Table S1b. Frequency and percentage of missing values in the variables of interest | | | | |
| --- | --- | --- | --- | --- |
| Variable | **Frequency of missing values** | **% of missing values** |  |  |
| Sleep Average | 927 | 2.8 |  |  |
| Sleep Weekday | 768 | 2.3 |  |  |
| Sleep Weekend | 808 | 2.4 |  |  |
| Restless Sleep | 819 | 2.4 |  |  |
| Enrollment Age | 0 | 0 |  |  |
| Enrollment Source | 0 | 0 |  |  |
| Race/Ethnicity | 307 | 0.9 |  |  |
| Education | 646 | 1.9 |  |  |
| Household Income | 836 | 2.5 |  |  |
| Employed | 700 | 2.1 |  |  |
| BMI | 669 | 2 |  |  |
| BPH | 628 | 1.9 |  |  |
| Depression | 458 | 1.4 |  |  |
| Hypertension | 450 | 1.3 |  |  |
| COPD | 468 | 1.4 |  |  |
| Asthma | 455 | 1.4 |  |  |
| Stroke | 473 | 1.4 |  |  |
| Alcohol Drinking | 899 | 2.7 |  |  |
| Smoking Status | 753 | 2.2 |  |  |
| Diabetes | 459 | 1.4 |  |  |
| Physical Activity | 1212 | 3.6 |  |  |

Missingness of exposure variables and covariates.

## **Table S2a. All baseline characteristics of men by sleep average in the SCCS, 2002-2009**

**Weighted Sleep Average Duration, hour**

|  | <6 Hours | 6 Hours | 7-8 Hours | ≥9 Hours | p-value^a^ |
| --- | --- | --- | --- | --- | --- |
| **Baseline Characteristic** | **N (%)** | **N (%)** | **N (%)** | **N (%)** |  |
|  |  |  |  |  |  |
| Enrollment age (years) |  |  |  |  | <.0001 |
| 40-49 | 2388 (51.2) | 3079 (47.4) | 6907 (44.2) | 2787 (47.6) |  |
| 50-59 | 1690 (36.3) | 2347 (36.1) | 5472 (35.0) | 1969 (33.6) |  |
| 60-69 | 480 (10.3) | 885 (13.6) | 2582 (16.5) | 860 (14.7) |  |
| 70-79 | 103 (2.2) | 191 (2.9) | 659 (4.2) | 242 (4.1) |  |
|  |  |  |  |  |  |
| Enrollment source |  |  |  |  | <.0001 |
| CHC in-person interview | 4310 (92.5) | 5501 (84.6) | 12511 (80.1) | 5179 (88.4) |  |
| General population | 351 (7.5) | 1001 (15.4) | 3109 (19.9) | 679 (11.6) |  |
|  |  |  |  |  |  |
| Race/Ethnicity |  |  |  |  | <.0001 |
| Non-Hispanic white | 1254 (27.0) | 1987 (30.8) | 5041 (32.5) | 1164 (20.0) |  |
| Non-Hispanic black | 3168 (68.2) | 4231 (65.5) | 9916 (63.9) | 4496 (77.1) |  |
| Other | 222 (4.8) | 242 (3.8) | 553 (3.6) | 171 (2.9) |  |
|  |  |  |  |  |  |
| Educational attainment |  |  |  |  | <.0001 |
| Less than 12 years | 1533 (33.0) | 1745 (27.0) | 4267 (27.5) | 2123 (36.5) |  |
| Graduated high school, training, or GED | 1870 (40.3) | 2510 (38.9) | 5945 (38.3) | 2300 (39.5) |  |
| Some college or junior college | 849 (18.3) | 1300 (20.1) | 2783 (18.0) | 909 (15.6) |  |
| Graduated college or beyond | 391 (8.4) | 905 (14.0) | 2513 (16.2) | 493 (8.5) |  |
|  |  |  |  |  |  |
| Annual household income |  |  |  |  | <.0001 |
| < $15,000 | 2873 (62.4) | 3290 (51.2) | 7618 (49.4) | 3689 (63.6) |  |
| $15,000 – $24,999 | 896 (19.4) | 1372 (21.4) | 3129 (20.3) | 1085 (18.7) |  |
| $25,000 – $49,999 | 549 (11.9) | 1015 (15.8) | 2291 (14.9) | 682 (11.8) |  |
| ≥$50,000 | 290 (6.3) | 747 (11.6) | 2375 (15.4) | 345 (6.0) |  |
|  |  |  |  |  |  |
| BMI (kg/m^2^) |  |  |  |  | <.0001 |
| <18.5 | 71 (1.5) | 79 (1.2) | 150 (0.97) | 76 (1.3) |  |
| 18.5 - <25.0 | 1501 (32.5) | 2003 (31.0) | 5028 (32.4) | 2046 (35.3) |  |
| 25.0 - <30.0 | 1515 (32.8) | 2319 (35.9) | 5807 (37.5) | 1930 (33.3) |  |
| ≥30.0 | 1531 (33.2) | 2068 (32.0) | 4514 (29.1) | 1750 (30.2) |  |
| Currently working |  |  |  |  | <.0001 |
| No | 3105 (67.1) | 3701 (57.3) | 8708 (56.1) | 4038 (69.5) |  |
| Yes | 1524 (32.9) | 2761 (42.7) | 6818 (43.9) | 1771 (30.5) |  |
|  |  |  |  |  |  |
| Smoking status |  |  |  |  | <.0001 |
| Never | 967 (20.9) | 1547 (24.0) | 3855 (24.9) | 1168 (20.1) |  |
| Former | 995 (21.5) | 1553 (24.1) | 4096 (26.5) | 1285 (22.1) |  |
| Current | 2673 (57.7) | 3355 (52.0) | 7505 (48.6) | 3360 (57.8) |  |
|  |  |  |  |  |  |
| Alcohol drinking status |  |  |  |  | 0.002 |
| Non-drinkers | 278 (6.0) | 391 (6.1) | 965 (6.3) | 352 (6.1) |  |
| Former drinkers | 1236 (26.8) | 1657 (25.8) | 3755 (24.3) | 1552 (26.8) |  |
| Current drinkers | 3095 (67.2) | 4383 (68.2) | 10714 (69.4) | 3890 (67.1) |  |
|  |  |  |  |  |  |
| Total physical activity (MET-hours/day), mean ± SD | 24.5 ± 23.6 | 26.0 ± 22.8 | 25.9 ± 22.3 | 24.1 ± 22.9 | <.0001 |
|  |  |  |  |  |  |
| HEI-2010, mean ± SD | 53.8 ± 11.5 | 55.1 ± 11.5 | 55.9 ± 11.6 | 54.5 ± 10.9 | <.0001 |
|  |  |  |  |  |  |
| History of diabetes |  |  |  |  | <.0001 |
| No | 3667 (78.7) | 5279 (81.2) | 12976 (83.1) | 4729 (80.8) |  |
| Yes | 992 (21.3) | 1220 (18.8) | 2632 (16.9) | 1127 (19.3) |  |
|  |  |  |  |  |  |
| History of depression |  |  |  |  | <.0001 |
| No | 3376 (72.5) | 5361 (82.5) | 13487 (86.4) | 4868 (83.1) |  |
| Yes | 1281 (27.5) | 1140 (17.5) | 2127 (13.6) | 987 (16.9) |  |
|  |  |  |  |  |  |
| History of hypertension |  |  |  |  | <.0001 |
| No | 2177 (46.8) | 3131 (48.2) | 8199 (52.5) | 2926 (50.0) |  |
| Yes | 2479 (53.2) | 3369 (51.8) | 7417 (47.5) | 2928 (50.0) |  |
|  |  |  |  |  |  |
| History of COPD |  |  |  |  | <.0001 |
| No | 4187 (90.0) | 6048 (93.1) | 14730 (94.4) | 5443 (93.0) |  |
| Yes | 468 (10.1) | 448 (6.9) | 880 (5.6) | 410 (7.0) |  |
|  |  |  |  |  |  |
| History of asthma |  |  |  |  | <.0001 |
| No | 3990 (85.7) | 5791 (89.1) | 14137 (90.5) | 5215 (89.1) |  |
| Yes | 667 (14.3) | 706 (10.9) | 1482 (9.5) | 641 (11.0) |  |
|  |  |  |  |  |  |
| History of stroke |  |  |  |  | <.0001 |
| No | 4266 (91.7) | 6133 (94.4) | 14783 (94.7) | 5431 (92.9) |  |
| Yes | 386 (8.3) | 362 (5.6) | 828 (5.3) | 418 (7.2) |  |
|  |  |  |  |  |  |
| Family history of PC |  |  |  |  | 0.002 |
| No | 4188 (95.1) | 5904 (94.7) | 13952 (94.2) | 5280 (95.5) |  |
| Yes | 218 (5.0) | 330 (5.3) | 855 (5.8) | 247 (4.5) |  |
|  |  |  |  |  |  |
| BPH |  |  |  |  | <.0001 |
| No | 4284 (92.4) | 5939 (91.8) | 14182 (91.2) | 5470 (93.8) |  |
| Yes | 355 (7.7) | 534 (8.3) | 1362 (8.8) | 362 (6.2) |  |
|  |  |  |  |  |  |
| PSA Screening |  |  |  |  | <.0001 |
| No | 2342 (53.8) | 3013 (49.5) | 7222 (48.9) | 3087 (55.7) |  |
| Yes | 2011 (46.2) | 3079 (50.5) | 7544 (51.1) | 2453 (44.3) |  |
|  |  |  |  |  |  |
| DRE screening |  |  |  |  | <.0001 |
| No | 1954 (42.2) | 2513 (38.9) | 6123 (39.4) | 2577 (44.2) |  |
| Yes | 2679 (57.8) | 3948 (61.1) | 9410 (60.6) | 3250 (55.8) |  |

Note: Some percentages may not equal to 100.0 due to rounding. Missing data not included in percentages.

^a^P-values represent Pearson Chi-square tests of association for categorical variables and Kruskal-Wallis tests for continuous variables.

SCCS: Southern Community Cohort Study. CHC: Community Health Center. BMI: body mass index.

SD: standard deviation. MET: metabolic equivalent of task. HEI-2010: Healthy Eating Index. COPD: Chronic obstructive

pulmonary disease. PC: prostate cancer. BPH: benign prostate hyperplasia. PSA: prostate cancer-specific antigen. DRE:

digital rectal examination.

## **Table S2b. All baseline characteristics of black men by sleep average in the SCCS, 2002-2009**

**Weighted Sleep Average Duration-Black Men, hour**

|  | <6 Hours | 6 Hours | 7-8 Hours | ≥9 Hours | p-value^a^ |
| --- | --- | --- | --- | --- | --- |
| **Baseline Characteristic** | **N (%)** | **N (%)** | **N (%)** | **N (%)** |  |
|  |  |  |  |  |  |
| Enrollment age (years) |  |  |  |  | <.0001 |
| 40-49 | 1642 (51.8) | 2136 (50.5) | 4989 (50.3) | 2305 (51.3) |  |
| 50-59 | 1165 (36.8) | 1539 (36.4) | 3434 (34.6) | 1513 (33.7) |  |
| 60-69 | 293 (9.3) | 461 (10.9) | 1200 (12.1) | 541 (12.0) |  |
| 70-79 | 68 (2.2) | 95 (2.3) | 293 (3.0) | 137 (3.1) |  |
|  |  |  |  |  |  |
| Enrollment source |  |  |  |  | <.0001 |
| CHC in-person interview | 2983 (94.2) | 3848 (91.0) | 9120 (92.0) | 4165 (92.6) |  |
| General population | 185 (5.8) | 383 (9.1) | 796 (8.0) | 331 (7.4) |  |
|  |  |  |  |  |  |
| Educational attainment |  |  |  |  | <.0001 |
| Less than 12 years | 1090 (34.4) | 1215 (28.7) | 3239 (32.7) | 1743 (38.8) |  |
| Graduated high school, training, or GED | 1258 (39.7) | 1715 (40.6) | 4113 (41.5) | 1828 (40.7) |  |
| Some college or junior college | 565 (17.9) | 860 (20.3) | 1681 (17.0) | 652 (14.5) |  |
| Graduated college or beyond | 253 (8.0) | 439 (10.4) | 881 (8.9) | 266 (5.9) |  |
|  |  |  |  |  |  |
| Annual household income |  |  |  |  | <.0001 |
| < $15,000 | 1966 (62.7) | 2327 (55.7) | 5684 (57.9) | 2986 (67.0) |  |
| $15,000 – $24,999 | 616 (19.7) | 926 (22.2) | 2230 (22.7) | 858 (19.2) |  |
| $25,000 – $49,999 | 381 (12.2) | 617 (14.8) | 1247 (12.7) | 456 (10.2) |  |
| ≥$50,000 | 172 (5.5) | 311 (7.4) | 657 (6.7) | 159 (3.6) |  |
|  |  |  |  |  |  |
| BMI (kg/m^2^) |  |  |  |  | <.0001 |
| <18.5 | 50 (1.6) | 53 (1.3) | 117 (1.2) | 56 (1.3) |  |
| 18.5 - <25.0 | 1061 (33.8) | 1420 (33.7) | 3438 (34.9) | 1691 (38.0) |  |
| 25.0 - <30.0 | 1038 (33.0) | 1484 (35.2) | 3562 (36.2) | 1489 (33.4) |  |
| ≥30.0 | 994 (31.6) | 1259 (29.9) | 2725 (27.7) | 1218 (27.4) |  |
|  |  |  |  |  |  |
| Currently working |  |  |  |  | <.0001 |
| No | 2047 (65.1) | 2408 (57.3) | 5808 (58.9) | 3051 (68.4) |  |
| Yes | 1098 (34.9) | 1795 (42.7) | 4048 (41.1) | 1409 (31.6) |  |
|  |  |  |  |  |  |
| Smoking status |  |  |  |  | <.0001 |
| Never | 709 (22.5) | 988 (23.5) | 2252 (22.8) | 857 (19.2) |  |
| Former | 642 (20.4) | 867 (20.6) | 2085 (21.1) | 841 (18.8) |  |
| Current | 1802 (57.2) | 2358 (56.0) | 5525 (56.0) | 2771 (62.0) |  |
|  |  |  |  |  |  |
| Alcohol drinking status |  |  |  |  | 0.063 |
| Non-drinkers | 177 (5.6) | 224 (5.4) | 576 (5.9) | 242 (5.4) |  |
| Former drinkers | 729 (23.2) | 1002 (23.9) | 2191 (22.3) | 1099 (24.7) |  |
| Current drinkers | 2232 (71.1) | 2964 (70.7) | 7054 (71.8) | 3114 (69.9) |  |
|  |  |  |  |  |  |
| Total physical activity (MET-hours/day), mean ± SD | 25.3 ± 23.4 | 26.3 ± 22.8 | 27.1 ± 23.4 | 25.4 ± 23.4 | <.0001 |
|  |  |  |  |  |  |
| HEI-2010, mean ± SD | 54.5 ± 11.3 | 55.0 ± 11.2 | 55.2 ± 11.0 | 54.6 ± 10.7 | 0.007 |
|  |  |  |  |  |  |
| History of diabetes |  |  |  |  | <.0001 |
| No | 2486 (78.5) | 3466 (82.0) | 8215 (82.9) | 3692 (82.2) |  |
| Yes | 681 (21.5) | 763 (18.0) | 1693 (17.1) | 802 (17.9) |  |
|  |  |  |  |  |  |
| History of depression |  |  |  |  | <.0001 |
| No | 2471 (78.1) | 3672 (86.8) | 8933 (90.1) | 3956 (88.0) |  |
| Yes | 694 (21.9) | 559 (13.2) | 977 (9.9) | 538 (12.0) |  |
| History of hypertension |  |  |  |  | <.0001 |
| No | 1479 (46.8) | 1987 (47.0) | 5090 (51.4) | 2280 (50.7) |  |
| Yes | 1684 (53.2) | 2242 (53.0) | 4823 (48.7) | 2214 (49.3) |  |
|  |  |  |  |  |  |
| History of COPD |  |  |  |  | <.0001 |
| No | 2946 (93.1) | 4024 (95.1) | 9492 (95.8) | 4264 (94.9) |  |
| Yes | 218 (6.9) | 207 (4.9) | 416 (4.2) | 228 (5.1) |  |
|  |  |  |  |  |  |
| History of asthma |  |  |  |  | <.0001 |
| No | 2752 (86.9) | 3804 (90.0) | 9021 (91.0) | 4044 (90.0) |  |
| Yes | 415 (13.1) | 422 (10.0) | 894 (9.0) | 450 (10.0) |  |
|  |  |  |  |  |  |
| History of stroke |  |  |  |  | <.0001 |
| No | 2936 (92.8) | 4008 (94.8) | 9411 (95.0) | 4214 (93.9) |  |
| Yes | 227 (7.2) | 218 (5.2) | 500 (5.0) | 276 (6.2) |  |
|  |  |  |  |  |  |
| Family history of PC |  |  |  |  | 0.036 |
| No | 2851 (95.0) | 3821 (94.6) | 8913 (94.9) | 4085 (95.9) |  |
| Yes | 150 (5.0) | 220 (5.4) | 477 (5.1) | 176 (4.1) |  |
|  |  |  |  |  |  |
| BPH |  |  |  |  | 0.0004 |
| No | 2960 (93.9) | 3956 (93.9) | 9379 (94.9) | 4286 (95.7) |  |
| Yes | 193 (6.1) | 257 (6.1) | 507 (5.1) | 195 (4.4) |  |
|  |  |  |  |  |  |
| PSA Screening |  |  |  |  | <.0001 |
| No | 1598 (53.5) | 2005 (50.4) | 5002 (53.2) | 2493 (58.4) |  |
| Yes | 1387 (46.5) | 1973 (49.6) | 4395 (46.8) | 1779 (41.6) |  |
|  |  |  |  |  |  |
| DRE screening |  |  |  |  | <.0001 |
| No | 1308 (41.5) | 1680 (40.0) | 4254 (43.1) | 2087 (46.7) |  |
| Yes | 1842 (58.5) | 2522 (60.0) | 5610 (56.9) | 2383 (53.3) |  |

Note: Some percentages may not equal to 100.0 due to rounding. Missing data not included in percentages.

^a^P-values represent Pearson Chi-square tests of association for categorical variables and Kruskal-Wallis tests for continuous variables.

SCCS: Southern Community Cohort Study. CHC: Community Health Center. BMI: body mass index.

SD: standard deviation. MET: metabolic equivalent of task. HEI-2010: Healthy Eating Index. COPD: Chronic obstructive

pulmonary disease. PC: prostate cancer. BPH: benign prostate hyperplasia. PSA: prostate cancer-specific antigen. DRE:

digital rectal examination.

## **Table S2c. All baseline characteristics of white men by sleep average in the SCCS, 2002-2009**

**Weighted Sleep Average Duration-White Men, hour**

|  | <6 Hours | 6 Hours | 7-8 Hours | ≥9 Hours | p-value^a^ |
| --- | --- | --- | --- | --- | --- |
| **Baseline Characteristic** | **N (%)** | **N (%)** | **N (%)** | **N (%)** |  |
|  |  |  |  |  |  |
| Enrollment age (years) |  |  |  |  | <.0001 |
| 40-49 | 632 (50.4) | 811 (40.8) | 1672 (33.2) | 395 (33.9) |  |
| 50-59 | 431 (34.4) | 712 (35.8) | 1797 (35.7) | 385 (33.1) |  |
| 60-69 | 159 (12.7) | 375 (18.9) | 1245 (24.7) | 286 (24.6) |  |
| 70-79 | 32 (2.6) | 89 (4.5) | 327 (6.5) | 98 (8.4) |  |
|  |  |  |  |  |  |
| Enrollment source |  |  |  |  | <.0001 |
| CHC in-person interview | 1125 (89.7) | 1468 (73.9) | 2995 (59.4) | 874 (75.1) |  |
| General population | 129 (10.3) | 519 (26.1) | 2046 (40.6) | 290 (24.9) |  |
|  |  |  |  |  |  |
| Educational attainment |  |  |  |  | <.0001 |
| Less than 12 years | 374 (29.9) | 484 (24.4) | 906 (18.0) | 333 (28.6) |  |
| Graduated high school, training, or GED | 538 (42.9) | 724 (36.5) | 1640 (32.6) | 408 (35.1) |  |
| Some college or junior college | 225 (18.0) | 377 (19.0) | 992 (19.7) | 219 (18.8) |  |
| Graduated college or beyond | 116 (9.3) | 401 (20.2) | 1498 (29.8) | 203 (17.5) |  |
|  |  |  |  |  |  |
| Annual household income |  |  |  |  | <.0001 |
| < $15,000 | 781 (62.8) | 851 (43.3) | 1690 (34.1) | 601 (52.3) |  |
| $15,000 – $24,999 | 226 (18.2) | 386 (19.7) | 776 (15.7) | 193 (16.8) |  |
| $25,000 – $49,999 | 137 (11.0) | 343 (17.5) | 932 (18.8) | 187 (16.3) |  |
| ≥$50,000 | 100 (8.0) | 384 (19.6) | 1557 (31.4) | 169 (14.7) |  |
|  |  |  |  |  |  |
| BMI (kg/m^2^) |  |  |  |  | <.0001 |
| <18.5 | 18 (1.5) | 23 (1.2) | 26 (0.5) | 17 (1.5) |  |
| 18.5 - <25.0 | 380 (30.6) | 516 (26.1) | 1413 (28.2) | 304 (26.3) |  |
| 25.0 - <30.0 | 394 (31.7) | 733 (37.1) | 1973 (39.4) | 371 (32.1) |  |
| ≥30.0 | 451 (36.3) | 703 (35.6) | 1598 (31.9) | 465 (40.2) |  |
|  |  |  |  |  |  |
| Currently working |  |  |  |  | <.0001 |
| No | 901 (72.3) | 1144 (57.9) | 2589 (51.6) | 851 (73.7) |  |
| Yes | 346 (27.8) | 832 (42.1) | 2425 (48.4) | 304 (26.3) |  |
|  |  |  |  |  |  |
| Smoking status |  |  |  |  | <.0001 |
| Never | 210 (16.8) | 481 (24.5) | 1417 (28.6) | 274 (23.7) |  |
| Former | 298 (23.9) | 609 (31.0) | 1797 (36.3) | 389 (33.6) |  |
| Current | 741 (59.3) | 876 (44.6) | 1741 (35.1) | 494 (42.7) |  |
|  |  |  |  |  |  |
| Alcohol drinking status |  |  |  |  | <.0001 |
| Non-drinkers | 83 (6.7) | 148 (7.5) | 340 (6.8) | 95 (8.3) |  |
| Former drinkers | 436 (35.1) | 584 (29.7) | 1400 (28.2) | 400 (34.8) |  |
| Current drinkers | 722 (58.2) | 1235 (62.8) | 3229 (65.0) | 656 (57.0) |  |
|  |  |  |  |  |  |
| Total physical activity (MET-hours/day), mean ± SD | 22.3 ± 23.5 | 25.6 ± 22.6 | 23.5 ± 19.7 | 19.3 ± 19.9 | <.0001 |
|  |  |  |  |  |  |
| HEI-2010, mean ± SD | 51.9 ± 11.5 | 54.8 ± 12.0 | 56.8 ± 12.6 | 54.1 ± 11.5 | <.0001 |
|  |  |  |  |  |  |
| History of diabetes |  |  |  |  | <.0001 |
| No | 1002 (80.0) | 1597 (80.4) | 4235 (84.1) | 893 (76.7) |  |
| Yes | 251 (20.0) | 389 (19.6) | 802 (15.9) | 271 (23.3) |  |
|  |  |  |  |  |  |
| History of depression |  |  |  |  | <.0001 |
| No | 755 (60.3) | 1481 (74.6) | 4003 (79.4) | 772 (66.4) |  |
| Yes | 498 (39.7) | 505 (25.4) | 1038 (20.6) | 391 (33.6) |  |
| History of hypertension |  |  |  |  | <.0001 |
| No | 593 (47.3) | 1000 (50.3) | 2744 (54.4) | 545 (46.9) |  |
| Yes | 661 (52.7) | 987 (49.7) | 2296 (45.6) | 617 (53.1) |  |
|  |  |  |  |  |  |
| History of COPD |  |  |  |  | <.0001 |
| No | 1031 (82.3) | 1770 (89.3) | 4632 (91.9) | 999 (85.9) |  |
| Yes | 222 (17.7) | 213 (10.7) | 407 (8.1) | 164 (14.1) |  |
|  |  |  |  |  |  |
| History of asthma |  |  |  |  | <.0001 |
| No | 1031 (82.4) | 1738 (87.5) | 4535 (90.0) | 993 (85.3) |  |
| Yes | 221 (17.7) | 249 (12.5) | 506 (10.0) | 171 (14.7) |  |
|  |  |  |  |  |  |
| History of stroke |  |  |  |  | <.0001 |
| No | 1124 (89.7) | 1861 (93.8) | 4753 (94.4) | 1031 (88.7) |  |
| Yes | 129 (10.3) | 124 (6.3) | 284 (5.6) | 131 (11.3) |  |
|  |  |  |  |  |  |
| Family history of PC |  |  |  |  | <.0001 |
| No | 1133 (95.9) | 1829 (95.5) | 4453 (92.9) | 1031 (94.4) |  |
| Yes | 48 (4.1) | 87 (4.5) | 343 (7.2) | 61 (5.6) |  |
|  |  |  |  |  |  |
| BPH |  |  |  |  | <.0001 |
| No | 1115 (89.2) | 1734 (87.7) | 4226 (84.4) | 1004 (86.8) |  |
| Yes | 135 (10.8) | 243 (12.3) | 781 (15.6) | 153 (13.2) |  |
|  |  |  |  |  |  |
| PSA Screening |  |  |  |  | <.0001 |
| No | 627 (54.5) | 884 (48.0) | 1926 (40.6) | 497 (45.9) |  |
| Yes | 524 (45.5) | 959 (52.0) | 2823 (59.4) | 586 (54.1) |  |
|  |  |  |  |  |  |
| DRE screening |  |  |  |  | <.0001 |
| No | 546 (43.8) | 735 (37.2) | 1632 (32.6) | 410 (35.4) |  |
| Yes | 702 (56.3) | 1240 (62.8) | 3380 (67.4) | 749 (64.6) |  |

Note: Some percentages may not equal to 100.0 due to rounding. Missing data not included in percentages.

^a^P-values represent Pearson Chi-square tests of association for categorical variables and Kruskal-Wallis tests for continuous variables.

SCCS: Southern Community Cohort Study. CHC: Community Health Center. BMI: body mass index.

SD: standard deviation. MET: metabolic equivalent of task. HEI-2010: Healthy Eating Index. COPD: Chronic obstructive

pulmonary disease. PC: prostate cancer. BPH: benign prostate hyperplasia. PSA: prostate cancer-specific antigen. DRE:

digital rectal examination.

## **Table S3. Complete analyses: Associations between sleep characteristics at enrollment and prostate cancer incidence, by prostate cancer aggressiveness in the SCCS**

|  | No Prostate Cancer (ref) | Non-Aggressiveness (Gleason Score (<8)) | | Aggressiveness (Gleason Score ≥ 8) | |
| --- | --- | --- | --- | --- | --- |
| Overall PC | n/N* | n/N* | OR (95% CI) ^a^ | n/N* | OR (95% CI) ^a^ |
|  |  |  |  |  |  |
| Sleep duration, hr |  |  |  |  |  |
| Sleep average |  |  |  |  |  |
| <6 | 4286/4388 | 81/4388 | 0.83 (0.65-1.07) | 21/4388 | 1.08 (0.66-1.77) |
| 6 | 5903/ 6094 | 158/6094 | 1.04 (0.86-1.26) | 33/ 6094 | 1.10 (0.73-1.67) |
| 7-8 | 14068/14528 | 384/14528 | ref | 76/14528 | ref |
| ≥9 | 5306/5480 | 141/5480 | 1.06 (0.87-1.30) | 33/5480 | 1.19 (0.79-1.81) |
|  |  |  | *p trend=0.18* |  | *p trend=0.87* |
|  |  |  |  |  |  |
| Weekday |  |  |  |  |  |
| <6 | 4671/4785 | 91/4785 | 0.79 (0.62-1.00) | 23/4785 | 1.03 (0.64-1.66) |
| 6 | 7181/7394 | 178/7394 | 0.89 (0.74-1.07) | 35/7394 | 0.93 (0.62-1.39) |
| 7-8 | 13797/14278 | 403/14278 | ref | 78/14278 | ref |
| ≥9 | 3986/4108 | 95/4108 | 0.86 (0.68-1.08) | 27/4108 | 1.22 (0.78-1.90) |
|  |  |  | *p trend=0.22* |  | *p trend=0.54* |
|  |  |  |  |  |  |
| Weekend |  |  |  |  |  |
| <6 | 4282/4382 | 78/4382 | 0.83 (0.64-1.06) | 22/4382 | 1.22 (0.74-1.99) |
| 6 | 5667/5850 | 149/5850 | 1.04 (0.86-1.27) | 34/5850 | 1.26 (0.83-1.92) |
| 7-8 | 13047/13482 | 368/13482 | ref | 67/13482 | ref |
| ≥9 | 6636/6847 | 171/6847 | 1.01 (0.84-1.22) | 40/6847 | 1.22 (0.82-1.82) |
|  |  |  | *p trend=0.32* |  | *p trend=0.75* |
|  |  |  |  |  |  |
| Restless sleep |  |  |  |  |  |
| Rarely/none of time | 10662/11057 | 321/11057 | ref | 74/11057 | ref |
| Some of the time | 11765/12114 | 298/12114 | 0.92 (0.78-1.09) | 51/12114 | 0.72 (0.50-1.04) |
| Much of the time | 3380/3470 | 72/3470 | 0.92 (0.71-1.21) | 18/3470 | 1.08 (0.64-1.84) |
| Most/all of the time | 3862/3955 | 74/3955 | 0.90 (0.69-1.17) | 19/3955 | 1.06 (0.63-1.79) |

| Note: Analysis is complete case only (thus observations with missing covariates or exposure of interest were excluded).  OR: odds ratio. 95% CI: 95% confidence interval  n= number of men with event for non-aggressiveness and aggressiveness columns but n=number of men without prostate cancer for ‘no prostate cancer’ reference column. N= Number of men within each group. |
| --- |
| ^a^ OR was adjusted for age at enrollment, race/ethnicity, enrollment source, education, income, employment status, BMI, depression, alcohol drinking, smoking status, diabetes, hypertension, COPD, asthma, stroke, total physical activity, and BPH  *The cohort and case numbers reflect the fully adjusted model numbers. |

## **Table S4. Multiple imputation: Associations between sleep characteristics at enrollment and overall prostate cancer incidence in the SCCS**

|  | Age and race/ethnicity adjusted^a^ | Fully adjusted^b^ |
| --- | --- | --- |
|  | HR (95% CI) | HR (95% CI) |
| Sleep duration, hr |  |  |
| Sleep average |  |  |
| <6 | 0.84 (0.70- 1.01) | 0.90 (0.74-1.08) |
| 6 | 1.02 (0.89-1.18) | 1.04 (0.90-1.20) |
| 7-8 | 1.00 (Ref) | 1.00 (Ref) |
| ≥9 | 1.06 (0.92-1.23) | 1.14 (0.99-1.33) |
|  | *p trend=0.06* | *p trend=0.05* |
|  |  |  |
| Weekday |  |  |
| <6 | 0.83 (0.70-0.99) | 0.87 (0.73-1.03) |
| 6 | 0.95 (0.83-1.09) | 0.95 (0.83-1.09) |
| 7-8 | 1.00 (Ref) | 1.00 (Ref) |
| ≥9 | 0.99 (0.84-1.16) | 1.06 (0.90-1.25) |
|  | *p trend=0.07* | *p trend=0.04* |
|  |  |  |
| Weekend |  |  |
| <6 | 0.81 (0.67-0.97) | 0.88 (0.73-1.06) |
| 6 | 1.01 (0.87-1.17) | 1.04 (0.90-1.21) |
| 7-8 | 1.00 (Ref) | 1.00 (Ref) |
| ≥9 | 1.03 (0.90-1.18) | 1.08 (0.94-1.24) |
|  | *p trend=0.05* | *p trend=0.12* |
|  |  |  |
| Restless sleep |  |  |
| Rarely/none of time | 1.00 (Ref) | 1.00 (Ref) |
| Some of the time | 0.94 (0.83-1.06) | 0.94 (0.83-1.06) |
| Much of the time | 0.95 (0.78-1.16) | 0.99 (0.81-1.20) |
| Most/all of the time | 0.90 (0.75-1.10) | 0.99 (0.81-1.20) |

HR: hazard ratio. 95% CI: 95% confidence interval

^a^ HR was adjusted for age at enrollment and race/ethnicity

^b^ HR was additionally adjusted for enrollment source, education, income, employment status,

BMI, depression, alcohol drinking, smoking status, diabetes, hypertension, COPD, asthma,

stroke, total physical activity, and BPH.

## **Table S5. Multiple imputation: Associations between sleep characteristics at enrollment and overall prostate cancer incidence by race in the SCCS**

|  | Age-adjusted^a^ | |  | Race/ethnicity-specific (fully adjusted)^b^ | | |
| --- | --- | --- | --- | --- | --- | --- |
|  | **Non-Hispanic Black** | **Non-Hispanic White** |  | **Non-Hispanic Black** | **Non-Hispanic White** | **p interaction** |
|  | HR (95% CI) | HR (95% CI) |  | HR (95% CI) | HR (95% CI) |  |
| Sleep duration, hr |  |  |  |  |  |  |
| Sleep average |  |  |  |  |  |  |
| <6 | 0.83 (0.67-1.02) | 0.88 (0.58-1.34) |  | 0.84 (0.68-1.04) | 1.16 (0.75-1.80) |  |
| 6 | 1.09 (0.93-1.29) | 0.84 (0.61-1.15) |  | 1.08 (0.91-1.27) | 0.95 (0.69-1.31) |  |
| 7-8 | 1.00 (Ref) | 1.00 (Ref) |  | 1.00 (Ref) | 1.00 (Ref) |  |
| ≥9 | 1.07 (0.91-1.26) | 1.08 (0.77-1.52) |  | 1.13 (0.96-1.33) | 1.25 (0.89-1.77) | *0.90* |
|  | *p trend=0.13* | *p trend=0.21* |  | *p trend=0.06* | *p trend=0.62* |  |
|  |  |  |  |  |  |  |
| Weekday |  |  |  |  |  |  |
| <6 | 0.84 (0.68-1.02) | 0.79 (0.52-1.20) |  | 0.84 (0.68-1.02) | 1.00 (0.65-1.54) |  |
| 6 | 0.98 (0.84-1.15) | 0.89 (0.66-1.18) |  | 0.96 (0.82-1.12) | 0.98 (0.73-1.32) |  |
| 7-8 | 1.00 (Ref) | 1.00 (Ref) |  | 1.00 (Ref) | 1.00 (Ref) |  |
| ≥9 | 0.98 (0.81-1.18) | 1.03 (0.71-1.48) |  | 1.03 (0.85-1.24) | 1.17 (0.80-1.71) |  |
|  | *p trend=0.19* | *p trend=0.20* |  | *p trend=0.08* | *p trend=0.59* | *0.94* |
|  |  |  |  |  |  |  |
| Weekend |  |  |  |  |  |  |
| <6 | 0.78 (0.63-0.97) | 0.89 (0.58-1.37) |  | 0.81 (0.65-1.01) | 1.21 (0.77-1.90) |  |
| 6 | 1.09 (0.92-1.29) | 0.76 (0.54-1.07) |  | 1.09 (0.92-1.30) | 0.90 (0.64-1.27) |  |
| 7-8 | 1.00 (Ref) | 1.00 (Ref) |  | 1.00 (Ref) | 1.00 (Ref) |  |
| ≥9 | 1.05 (0.90-1.23) | 0.96 (0.71-1.31) |  | 1.09 (0.93-1.28) | 1.05 (0.77-1.43) | *0.79* |
|  | *p trend=0.08* | *p trend=0.34* |  | *p trend=0.08* | *p trend=0.96* |  |
|  |  |  |  |  |  |  |
| Restless sleep |  |  |  |  |  |  |
| Rarely/none of time | 1.00 (Ref) | 1.00 (Ref) |  | 1.00 (Ref) | 1.00 (Ref) |  |
| Some of the time | 0.89 (0.78-1.03) | 1.10 (0.84-1.43) |  | 0.91 (0.79-1.05) | 1.06 (0.81-1.39) |  |
| Much of the time | 0.89 (0.70-1.14) | 1.19 (0.82-1.74) |  | 0.93 (0.73-1.18) | 1.26 (0.86-1.85) |  |
| Most/all of the time | 0.88 (0.71-1.11) | 1.03 (0.69-1.53) |  | 0.94 (0.75-1.19) | 1.22 (0.81-1.85) | *0.17* |

Among those who are black and white only. Due to small sample size, ‘Other’ race/ethnicity was excluded.

HR: hazard ratio. 95% CI: 95% confidence interval

^a^ HR was adjusted for age at enrollment

^b^ HR was additionally adjusted for enrollment source, education, income, employment status, BMI, depression, alcohol drinking, smoking status, diabetes, hypertension, COPD, asthma, stroke, total physical activity, and BPH.

## **Table S6. Associations between sleep characteristics at enrollment and overall prostate cancer incidence in the SCCS**

|  | Cohort N=33,319 | Cases N=1,096 | Age and race/ethnicity adjusted^a^ | Fully adjusted^b^ |
| --- | --- | --- | --- | --- |
|  | Overall | Overall | Overall | Overall |
|  | N (%)* | N (%)* | HR (95% CI) | HR (95% CI) |
| Sleep duration, hr |  |  |  |  |
| Sleep average |  |  |  |  |
| <6 | 4370 (14.4) | 104 (10.7) | 0.83 (0.68- 1.02) | 0.85 (0.68-1.05) |
| 6 | 6055 (20.0) | 202 (20.7) | 1.01 (0.86-1.19) | 1.05 (0.89-1.24) |
| 7-8 | 14421 (47.6) | 484 (49.7) | 1.00 (Ref) | 1.00 (Ref) |
| ≥9 | 5437 (18.0) | 184 (18.9) | 1.04 (0.88-1.22) | 1.10 (0.93-1.31) |
|  |  |  | *p trend=0.11* | *p trend=0.09* |
|  |  |  |  |  |
| Weekday |  |  |  |  |
| <6 | 4764 (15.7) | 114 (11.7) | 0.81 (0.67-0.98) | 0.79 (0.64-0.97) |
| 6 | 7354 (24.2) | 231 (23.6) | 0.97 (0.83-1.12) | 0.94 (0.80-1.10) |
| 7-8 | 14168 (46.7) | 502 (51.4) | 1.00 (Ref) | 1.00 (Ref) |
| ≥9 | 4072 (13.4) | 130 (13.3) | 0.94 (0.79-1.13) | 0.95 (0.79-1.16) |
|  |  |  | *p trend=0.16* | *p trend=0.08* |
|  |  |  |  |  |
| Weekend |  |  |  |  |
| <6 | 4363 (14.4) | 98 (10.1) | 0.80 (0.65-0.98) | 0.83 (0.66-1.03) |
| 6 | 5819 (19.2) | 198 (20.3) | 1.05 (0.89-1.23) | 1.10 (0.93-1.31) |
| 7-8 | 13373 (44.1) | 454 (46.6) | 1.00 (Ref) | 1.00 (Ref) |
| ≥9 | 6798 (22.4) | 225 (23.1) | 1.03 (0.89-1.20) | 1.06 (0.90-1.24) |
|  |  |  | *p trend=0.10* | *p trend=0.20* |
|  |  |  |  |  |
| Restless sleep | |  |  |  |
| Rarely/none of time | 10975 (36.1) | 414 (42.6) | 1.00 (Ref) | 1.00 (Ref) |
| Some of the time | 12031 (39.6) | 369 (38.0) | 0.90 (0.79-1.03) | 0.89 (0.77-1.02) |
| Much of the time | 3451 (11.4) | 88 (9.1) | 0.90 (0.72-1.12) | 0.90 (0.71-1.13) |
| Most/all of the time | 3932 (12.9) | 100 (10.3) | 0.86 (0.69-1.06) | 0.94 (0.75-1.18) |

Note: Excluding cases diagnosed within 2 years after sleep assessment. Analysis is complete case only (thus observations with missing covariates or exposure of interest were excluded).

HR: hazard ratio. 95% CI: 95% confidence interval.

^a^ HR was adjusted for age at enrollment and race/ethnicity

^b^ HR was additionally adjusted for enrollment source, education, income, employment status, BMI, depression, alcohol drinking, smoking status, diabetes, hypertension, COPD, asthma, stroke, total physical activity, and BPH.

*The cohort and case numbers reflect the fully adjusted model numbers.

Of the 33,319 participants in the cohort after excluding men diagnosed with prostate cancer within 2 years of follow up, 2,809 were excluded from the fully adjusted models for each exposure due to any missing covariate data.

## **Table S7. Associations between sleep characteristics at enrollment and prostate cancer incidence by race in the SCCS (Cohort N=31,810)**

|  | Cases (Black) N=808 | Cases (White) N=244 | Age-adjusted^a^ | |  | Race/ethnicity-specific (fully adjusted)^b^ | | |
| --- | --- | --- | --- | --- | --- | --- | --- | --- |
|  |  |  | **Non-Hispanic Black** | **Non-Hispanic White** |  | **Non-Hispanic Black** | **Non-Hispanic White** | **p interaction** |
|  | N (%)* | N (%)* | HR (95% CI) | HR (95% CI) |  | HR (95% CI) | HR (95% CI) |  |
| Sleep duration, hr |  |  |  |  |  |  |  |  |
| Sleep average |  |  |  |  |  |  |  |  |
| <6 | 78 (10.5) | 23 (11.1) | 0.80 (0.64-1.01) | 1.00 (0.64-1.56) |  | 0.76 (0.59-0.97) | 1.43 (0.89-2.29) |  |
| 6 | 162 (21.8) | 35 (16.9) | 1.09 (0.91-1.31) | 0.82 (0.57-1.17) |  | 1.09 (0.91-1.32) | 0.94 (0.64-1.37) |  |
| 7-8 | 350 (47.2) | 120 (58.0) | 1.00 (Ref) | 1.00 (Ref) |  | 1.00 (Ref) | 1.00 (Ref) |  |
| ≥9 | 152 (20.5) | 29 (14.0) | 1.02 (0.85-1.23) | 1.21 (0.84-1.74) |  | 1.07 (0.89-1.30) | 1.29 (0.85-1.96) | 0.58 |
|  |  |  | *p trend=0.22* | *p trend=0.25* |  | *p trend=0.07* | *p trend=0.89* |  |
|  |  |  |  |  |  |  |  |  |
| Weekday |  |  |  |  |  |  |  |  |
| <6 | 88 (11.8) | 23 (11.1) | 0.79 (0.64-0.99) | 0.90 (0.57-1.40) |  | 0.73 (0.57-0.92) | 1.21 (0.76-1.94) |  |
| 6 | 181 (24.3) | 45 (21.6) | 0.99 (0.83-1.18) | 0.92 (0.67-1.27) |  | 0.94 (0.78-1.12) | 1.00 (0.70-1.41) |  |
| 7-8 | 370 (49.7) | 118 (56.7) | 1.00 (Ref) | 1.00 (Ref) |  | 1.00 (Ref) | 1.00 (Ref) |  |
| ≥9 | 105 (14.1) | 22 (10.6) | 0.90 (0.73-1.11) | 1.16 (0.77-1.73) |  | 0.91 (0.73-1.13) | 1.14 (0.72-1.83) |  |
|  |  |  | *p trend=0.32* | *p trend=0.31* |  | *p trend=0.07* | *p trend=0.81* | *0.42* |
|  |  |  |  |  |  |  |  |  |
| Weekend |  |  |  |  |  |  |  |  |
| <6 | 73 (9.8) | 21 (10.1) | 0.76 (0.60-0.97) | 0.98 (0.62-1.56) |  | 0.74 (0.57-0.95) | 1.40 (0.86-2.30) |  |
| 6 | 163 (22.0) | 31 (14.9) | 1.14 (0.95-1.37) | 0.76 (0.52-1.11) |  | 1.17 (0.97-1.41) | 0.93 (0.62-1.39) |  |
| 7-8 | 319 (43.0) | 122 (58.7) | 1.00 (Ref) | 1.00 (Ref) |  | 1.00 (Ref) | 1.00 (Ref) |  |
| ≥9 | 187 (25.2) | 34 (16.4) | 1.04 (0.87-1.24) | 1.04 (0.74-1.45) |  | 1.08 (0.90-1.29) | 0.99 (0.68-1.46) | *0.29* |
|  |  |  | *p trend=0.16* | *p trend=0.38* |  | p trend=0.09 | *p trend=0.43* |  |
| Restless sleep | |  |  |  |  |  |  |  |
| Rarely/none of time | 337 (45.4) | 65 (31.7) | 1.00 (Ref) | 1.00 (Ref) |  | 1.00 (Ref) | 1.00 (Ref) |  |
| Some of the time | 277 (37.3) | 86 (42.0) | 0.83 (0.71-0.97) | 1.20 (0.89-1.62) |  | 0.84 (0.72-0.99) | 1.18 (0.86-1.64) |  |
| Much of the time | 58 (7.8) | 27 (13.2) | 0.81 (0.62-1.06) | 1.26 (0.83-1.92) |  | 0.80 (0.61-1.07) | 1.32 (0.83-2.09) |  |
| Most/all of the time | 70 (9.4) | 27 (13.2) | 0.81 (0.63-1.05) | 1.14 (0.74-1.77) |  | 0.85 (0.65-1.11) | 1.42 (0.88-2.30) | *0.03* |

Note: Excluding cases diagnosed within 2 years after sleep assessment. Among those who are black and white only (n=31,810). Due to small sample size, ‘Other’ race (1205), was excluded. Analysis is complete case only (thus observations with missing covariates or exposure of interest were excluded).

HR: hazard ratio. 95% CI: 95% confidence interval

^a^ HR was adjusted for age at enrollment

^b^ HR was additionally adjusted for enrollment source, education, income, employment status, BMI, depression, alcohol drinking, smoking status, diabetes, hypertension, COPD, asthma, stroke, total physical activity, and BPH.

*The case numbers reflect the fully adjusted model numbers.

Of the 22,261 black participants and 9,549 white participants in the analytic cohort after excluding men diagnosed with prostate cancer within 2 years of follow up, 1,542 were excluded from the fully adjusted models for each exposure due to any missing covariate data for black men, and 840 were excluded from the fully adjusted models for each exposure due to any missing covariate data for white men.

## **Table S8. Complete analyses: Associations between sleep characteristics at enrollment and overall prostate cancer incidence in the SCCS**

|  | Cohort N=33,568 | Cases N=1345 | Age and race/ethnicity adjusted^a^ | Fully adjusted^b^ |
| --- | --- | --- | --- | --- |
|  | N (%)* | N (%)* | HR (95% CI) | HR (95% CI) |
| Sleep duration, hr |  |  |  |  |
| Sleep average |  |  |  |  |
| <6 | 3592 (14.0) | 105 (10.4) | 0.82 (0.69- 0.99) | 0.86 (0.69-1.06) |
| 6 | 5219 (20.3) | 210 (20.8) | 1.02 (0.88-1.18) | 1.05 (0.89-1.24) |
| 7-8 | 12393 (48.2) | 507 (50.1) | 1.00 (Ref) | 1.00 (Ref) |
| ≥9 | 4520 (17.6) | 190 (18.8) | 1.06 (0.92-1.23) | 1.12 (0.95-1.33) |
|  |  |  | *p trend=0.04* | *p trend=0.09* |
|  |  |  |  |  |
| Weekday |  |  |  |  |
| <6 | 3957 (15.4) | 117 (11.5) | 0.81 (0.68-0.97) | 0.81 (0.66-1.00) |
| 6 | 6331 (24.6) | 238 (23.5) | 0.95 (0.83-1.09) | 0.94 (0.81-1.10) |
| 7-8 | 12135 (47.1) | 524 (51.6) | 1.00 (Ref) | 1.00 (Ref) |
| ≥9 | 3355 (13.0) | 136 (13.4) | 0.99 (0.84-1.16) | 0.97 (0.80-1.18) |
|  |  |  | *p trend=0.05* | *p trend=0.10* |
|  |  |  |  |  |
| Weekend |  |  |  |  |
| <6 | 3580 (13.9) | 100 (9.9) | 0.80 (0.66-0.96) | 0.84 (0.68-1.05) |
| 6 | 4964 (19.3) | 197 (19.5) | 1.00 (0.86-1.16) | 1.05 (0.89-1.24) |
| 7-8 | 11512 (44.7) | 482 (47.6) | 1.00 (Ref) | 1.00 (Ref) |
| ≥9 | 5721 (22.2) | 234 (23.1) | 1.03 (0.90-1.18) | 1.07 (0.91-1.25) |
|  |  |  | *p trend=0.04* | *p trend=0.13* |
|  |  |  |  |  |
| Restless sleep | |  |  |  |
| Rarely/none of time | 9497 (36.8) | 426 (42.1) | 1.00 (Ref) | 1.00 (Ref) |
| Some of the time | 10219 (39.6) | 389 (38.4) | 0.94 (0.83-1.06) | 0.95 (0.83-1.09) |
| Much of the time | 2905 (11.3) | 94 (9.3) | 0.94 (0.77-1.15) | 0.98 (0.78-1.23) |
| Most/all of the time | 3184 (12.3) | 104 (10.3) | 0.90 (0.75-1.10) | 1.04 (0.83-1.30) |

HR: hazard ratio. 95% CI: 95% confidence interval

^a^ HR was adjusted for age at enrollment and race/ethnicity

^b^ HR was additionally adjusted for enrollment source, education, income, employment status, BMI, depression, alcohol drinking, smoking status, diabetes, hypertension, COPD, asthma, stroke, total physical activity, HEI-2010, family history (father and brother) of prostate cancer, BPH, PSA and DRE screening

*The cohort and case numbers reflect the fully adjusted model numbers.

Of the 33,568 participants in the analytic cohort, 7,677 were excluded from the fully adjusted models for each exposure due to any missing covariate data.

## **Table S9. Complete analyses: Associations between sleep characteristics at enrollment and prostate cancer incidence by race in the SCCS (Cohort N=32,046)**

|  | Cases (Black) N=984 | Cases (White) N=304 | Age-adjusted^a^ | |  | Race/ethnicity-specific (fully adjusted)^b^ | | |
| --- | --- | --- | --- | --- | --- | --- | --- | --- |
|  |  |  | **Non-Hispanic Black** | **Non-Hispanic White** |  | **Non-Hispanic Black** | **Non-Hispanic White** | **p interaction** |
|  | N (%)* | N (%)* | HR (95% CI) | HR (95% CI) |  | HR (95% CI) | HR (95% CI) |  |
| Sleep duration, hr |  |  |  |  |  |  |  |  |
| Sleep average |  |  |  |  |  |  |  |  |
| <6 | 78 (10.4) | 23 (10.0) | 0.81 (0.66-1.00) | 0.86 (0.57-1.32) |  | 0.78 (0.61-1.00) | 1.29 (0.81-2.05) |  |
| 6 | 162 (21.5) | 42 (18.2) | 1.09 (0.92-1.29) | 0.83 (0.61-1.14) |  | 1.08 (0.90-1.30) | 1.01 (0.71-1.43) |  |
| 7-8 | 354 (47.1) | 138 (59.7) | 1.00 (Ref) | 1.00 (Ref) |  | 1.00 (Ref) | 1.00 (Ref) |  |
| ≥9 | 158 (21.0) | 28 (12.1) | 1.07 (0.91-1.27) | 1.09 (0.78-1.53) |  | 1.12 (0.92-1.35) | 1.14 (0.75-1.74) | 0.32 |
|  |  |  | *p trend=0.10* | *p trend=0.17* |  | *p trend=0.05* | *p trend=0.71* |  |
|  |  |  |  |  |  |  |  |  |
| Weekday |  |  |  |  |  |  |  |  |
| <6 | 90 (11.9) | 23 (9.9) | 0.82 (0.67-1.00) | 0.77 (0.50-1.17) |  | 0.76 (0.61-0.96) | 1.08 (0.68-1.72) |  |
| 6 | 181 (24.0) | 51 (22.0) | 0.98 (0.83-1.15) | 0.88 (0.66-1.18) |  | 0.95 (0.79-1.13) | 1.00 (0.72-1.39) |  |
| 7-8 | 371 (49.2) | 138 (59.5) | 1.00 (Ref) | 1.00 (Ref) |  | 1.00 (Ref) | 1.00 (Ref) |  |
| ≥9 | 112 (14.9) | 20 (8.6) | 0.98 (0.82-1.18) | 1.03 (0.72-1.49) |  | 0.96 (0.77-1.19) | 0.96 (0.59-1.55) | *0.29* |
|  |  |  | *p trend=0.14* | *p trend=0.16* |  | *p trend=0.08* | *p trend=0.71* |  |
|  |  |  |  |  |  |  |  |  |
| Weekend |  |  |  |  |  |  |  |  |
| <6 | 73 (9.7) | 22 (9.5) | 0.77 (0.62-0.96) | 0.89 (0.58-1.36) |  | 0.74 (0.58-0.96) | 1.33 (0.83-2.15) |  |
| 6 | 157 (20.9) | 35 (15.1) | 1.08 (0.91-1.28) | 0.75 (0.53-1.05) |  | 1.10 (0.91-1.33) | 0.96 (0.66-1.40) |  |
| 7-8 | 329 (43.8) | 139 (59.9) | 1.00 (Ref) | 1.00 (Ref) |  | 1.00 (Ref) | 1.00 (Ref) |  |
| ≥9 | 193 (25.7) | 36 (15.5) | 1.05 (0.90-1.23) | 0.96 (0.71-1.31) |  | 1.09 (0.91-1.31) | 0.98 (0.68-1.42) | *0.19* |
|  |  |  | *p trend=0.06* | *p trend=0.30* |  | *p trend=0.04* | *p trend=0.43* |  |
|  |  |  |  |  |  |  |  |  |
| continued | **Cases (Black) N=984** | **Cases (White) N=304** | **Age-adjusted^a^** | |  | **Race/ethnicity-specific (fully adjusted)^b^** | | |
|  |  |  | **Non-Hispanic Black** | **Non-Hispanic White** |  | **Non-Hispanic Black** | **Non-Hispanic White** | **p interaction** |
|  | N (%)^*^ | N (%)^*^ | HR (95% CI) | HR (95% CI) |  | HR (95% CI) | HR (95% CI) |  |
| Restless sleep | |  |  |  |  |  |  |  |
| Rarely/none of time | 336 (44.6) | 77 (33.3) | 1.00 (Ref) | 1.00 (Ref) |  | 1.00 (Ref) | 1.00 (Ref) |  |
| Some of the time | 288 (38.2) | 93 (40.3) | 0.90 (0.78-1.03) | 1.11 (0.85-1.44) |  | 0.93 (0.79-1.09) | 1.10 (0.81-1.49) |  |
| Much of the time | 60 (8.0) | 31 (13.4) | 0.88 (0.69-1.12) | 1.19 (0.82-1.74) |  | 0.89 (0.67-1.18) | 1.30 (0.85-2.00) |  |
| Most/all of the time | 70 (9.3) | 30 (13.0) | 0.88 (0.70-1.11) | 1.04 (0.70-1.54) |  | 0.95 (0.73-1.24) | 1.42 (0.91-2.23) | *0.05* |

Note: Among those who are black and white only (n=32,046). Due to small sample size, ‘Other’ race (n=1215), was excluded. Analysis is complete case only (thus observations with missing covariates or exposure of interest were excluded).

HR: hazard ratio. 95% CI: 95% confidence interval

^a^ HR was adjusted for age at enrollment

^b^ HR was additionally adjusted for enrollment source, education, income, employment status, BMI, depression, alcohol drinking, smoking status, diabetes, hypertension, COPD, asthma, stroke, total physical activity, HEI-2010, family history (father and brother) of prostate cancer, BPH, PSA and DRE screening

*The case numbers reflect the fully adjusted model numbers.

Of the 22,437 black participants and 9,609 white participants in the analytic cohort, 5,089 were excluded from the fully adjusted models for each exposure due to any missing covariate data for black men, and 1,996 were excluded from the fully adjusted models for each exposure due to any missing covariate data for white men.

## **Table S10. Complete analyses: Associations between sleep characteristics at enrollment and prostate cancer incidence, by prostate cancer aggressiveness in the SCCS**

|  | No Prostate Cancer (ref) | Non-Aggressiveness (Gleason Score (<8)) | | Aggressiveness (Gleason Score ≥ 8) | |
| --- | --- | --- | --- | --- | --- |
| Overall PC | n/N* | n/N* | OR (95% CI) ^a^ | n/N* | OR (95% CI) ^a^ |
|  |  |  |  |  |  |
| Sleep duration, hr |  |  |  |  |  |
| Sleep average |  |  |  |  |  |
| <6 | 3502/3592 | 70/3592 | 0.87 (0.66-1.13) | 20/3592 | 1.34 (0.80-2.25) |
| 6 | 5049/5219 | 142/5219 | 1.09 (0.89-1.34) | 28/5219 | 1.18 (0.75-1.86) |
| 7-8 | 12002/12393 | 330/12393 | ref | 61/12393 | Ref |
| ≥9 | 4372/4520 | 119/4520 | 1.09 (0.87-1.36) | 29/4520 | 1.37 (0.87-2.15) |
|  |  |  | *p trend=0.34* |  | *p trend=0.85* |
|  |  |  |  |  |  |
| Weekday |  |  |  |  |  |
| <6 | 3856/3957 | 79/3957 | 0.81 (0.63-1.05) | 22/3957 | 1.29 (0.78-2.12) |
| 6 | 6141/6331 | 159/6331 | 0.93 (0.77-1.13) | 31/ 6331 | 1.05 (0.68-1.62) |
| 7-8 | 11727/12135 | 346/12135 | ref | 62/12135 | ref |
| ≥9 | 3252/3355 | 80/3355 | 0.87 (0.68-1.13) | 23/3355 | 1.35 (0.83-2.20) |
|  |  |  | *p trend=0.39* |  | *p trend=0.94* |
|  |  |  |  |  |  |
| Weekend |  |  |  |  |  |
| <6 | 3492/3580 | 68/3580 | 0.87 (0.67-1.15) | 20/3580 | 1.50 (0.88-2.55) |
| 6 | 4802/4964 | 132/4964 | 1.08 (0.88-1.34) | 30/4964 | 1.48 (0.94-2.33) |
| 7-8 | 11144/11512 | 316/11512 | ref | 52/11512 | ref |
| ≥9 | 5539/5721 | 146/5721 | 1.03 (0.84-1.26) | 36/5721 | 1.47 (0.95-2.26) |
|  |  |  | *p trend=0.54* |  | *p trend=0.62* |
|  |  |  |  |  |  |
| Restless sleep |  |  |  |  |  |
| Rarely/none of time | 9158/9497 | 274/9497 | ref | 65/9497 | ref |
| Some of the time | 9915/10219 | 264/10219 | 0.99 (0.83-1.18) | 40/10219 | 0.65 (0.44-0.97) |
| Much of the time | 2825/2905 | 64/2905 | 0.99 (0.75-1.32) | 16/2905 | 1.11 (0.63-1.95) |
| Most/all of the time | 3105/3184 | 62/3184 | 0.94 (0.70-1.26) | 17/3184 | 1.13 (0.65-1.98) |

| Note: Analysis is complete case only (thus observations with missing covariates or exposure of interest were excluded).  OR: odds ratio. 95% CI: 95% confidence interval  n= number of men with event for non-aggressiveness and aggressiveness columns but n=number of men without prostate cancer for ‘no prostate cancer’ reference column; N= Number of men within each group. |
| --- |
| ^a^ OR was adjusted for age at enrollment, race/ethnicity, enrollment source, education, income, employment status, BMI, depression, alcohol drinking, smoking status, diabetes, hypertension, COPD, asthma, stroke, total physical activity, Hei-2010, family history (father and brother) of prostate cancer, BPH, PSA and DRE screening  *The cohort and case numbers reflect the fully adjusted model numbers. |
|  |

## **Table S11. Complete analyses: Associations between sleep characteristics at enrollment and prostate cancer incidence, by prostate cancer aggressiveness and race in the SCCS**

|  | Non-Aggressiveness^a^-Black Men | | Aggressiveness^b^-Black Men | |  | Non-Aggressiveness-White Men | | Aggressiveness-White Men | |
| --- | --- | --- | --- | --- | --- | --- | --- | --- | --- |
| Overall PC | n/N* | OR (95% CI) ^c^ | n/N* | OR (95% CI) ^c^ |  | n/N* | OR (95% CI) ^c^ | n/N* | OR (95% CI) ^c^ |
| Sleep duration, hr |  |  |  |  |  |  |  |  |  |
| Sleep average |  |  |  |  |  |  |  |  |  |
| <6 | 52/2453 | 0.77 (0.57-1.05) | 15/2453 | 1.30 (0.72-2.35) |  | 15/982 | 1.31 (0.73-2.36) | 5/982 | 1.72 (0.57-5.16) |
| 6 | 112/3409 | 1.13 (0.90-1.43) | 20/3409 | 1.12 (0.66-1.92) |  | 27/1616 | 0.99 (0.64-1.55) | 6/1616 | 1.19 (0.45-3.16) |
| 7-8 | 228/7890 | ref | 44/7890 | ref |  | 92/4057 | ref | 14/4057 | ref |
| ≥9 | 98/3491 | 1.09 (0.85-1.39) | 26/3491 | 1.48 (0.91-2.43) |  | 18/902 | 1.19 (0.70-2.03) | 3/902 | 0.90 (0.25-3.24) |
|  |  | *p trend=0.20* |  | *p trend=0.75* |  |  | *p trend=0.82* |  | *p trend=0.32* |
|  |  |  |  |  |  |  |  |  |  |
| Weekday |  |  |  |  |  |  |  |  |  |
| <6 | 61 /2709 | 0.74 (0.56-0.99) | 17/2709 | 1.30 (0.74-2.30) |  | 15/1076 | 1.10 (0.61-1.97) | 5/1076 | 1.42 (0.48-4.19) |
| 6 | 123/4195 | 0.93 (0.74-1.16) | 23/4195 | 1.03 (0.62-1.72) |  | 33/1902 | 1.00 (0.66-1.51) | 6/1902 | 0.92 (0.35-2.43) |
| 7-8 | 244/7808 | ref | 44/7808 | ref |  | 92/3899 | ref | 15/3899 | ref |
| ≥9 | 64/2565 | 0.84 (0.64-1.12) | 21/2565 | 1.54 (0.91-2.62) |  | 13/698 | 0.99 (0.54-1.83) | 2/698 | 0.63 (0.14-2.86) |
|  |  | *p trend=0.28* |  | *p trend=0.74* |  |  | *p trend=0.81* |  | *p trend=0.42* |
|  |  |  |  |  |  |  |  |  |  |
| Weekend |  |  |  |  |  |  |  |  |  |
| <6 | 50/2482 | 0.77 (0.56-1.06) | 14/2482 | 1.33 (0.71-2.48) |  | 15/942 | 1.39 (0.77-2.53) | 5/942 | 1.86 (0.61-5.67) |
| 6 | 108/3360 | 1.15 (0.91-1.46) | 22/3360 | 1.43 (0.84-2.43) |  | 22/1422 | 0.93 (0.57-1.51) | 6/1422 | 1.38 (0.51-3.73) |
| 7-8 | 213/7154 | ref | 37/7154 | ref |  | 93/3947 | ref | 13/3947 | ref |
| ≥9 | 119/4279 | 1.05 (0.83-1.32) | 32/4279 | 1.60 (0.99-2.58) |  | 23/1267 | 0.99 (0.61-1.59) | 4/1267 | 0.91 (0.29-2.88) |
|  |  | *p trend=0.31* |  | *p trend=0.76* |  |  | *p trend=0.50* |  | *p trend=0.23* |
|  |  |  |  |  |  |  |  |  |  |
| Restless sleep |  |  |  |  |  |  |  |  |  |
| Rarely/none of time | 214/6684 | ref | 52/6684 | ref |  | 51/2496 | ref | 11/2496 | ref |
| Some of the time | 195/7041 | 0.97 (0.79-1.19) | 33/7041 | 0.71 (0.46-1.10) |  | 64/2850 | 1.16 (0.79-1.69) | 6/2850 | 0.51 (0.18-1.40) |
| Much of the time | 42/1674 | 0.93 (0.66-1.31) | 8/1674 | 0.81 (0.38-1.73) |  | 21/1103 | 1.31 (0.76-2.23) | 6/1103 | 1.44 (0.50-4.15) |
| Most/all of the time | 40/1912 | 0.80 (0.56-1.14) | 12/1912 | 1.20 (0.63-2.29) |  | 19/1120 | 1.46 (0.82-2.59) | 5/1120 | 1.16 (0.37-3.63) |

Note: Analysis is complete case only (thus observations with missing covariates or exposure of interest were excluded). n= number of men with event for non-aggressiveness and aggressiveness columns but n=number of men without prostate cancer for ‘no prostate cancer’ reference column; N= Number of men within each group.

OR: odds ratio. 95% CI: 95% confidence interval

^a^Non-aggressiveness defined as Gleason score <8.

^b^Aggressiveness defined as Gleason score ≥ 8.

^c^ OR was adjusted for age at enrollment, enrollment source, education, income, employment status, BMI, depression, alcohol drinking, smoking status, diabetes, hypertension, COPD, asthma, stroke, total physical activity, HEI-2010, family history (father and brother) of prostate cancer, BPH, PSA and DRE screening

*The cohort and case numbers reflect the fully adjusted model numbers.
